# Inferring Fine‐Scale Mutation and Recombination Rate Maps in Aye‐Ayes (*Daubentonia madagascariensis*)

**DOI:** 10.1002/ece3.72314

**Published:** 2025-11-03

**Authors:** Vivak Soni, Cyril J. Versoza, John W. Terbot, Jeffrey D. Jensen, Susanne P. Pfeifer

**Affiliations:** ^1^ Center for Evolution and Medicine, School of Life Sciences Arizona State University Tempe Arizona USA

**Keywords:** fine‐scale mapping, mutation, population genomics, primate, recombination, strepsirrhine

## Abstract

The rate of input of new genetic mutations, and the rate at which that variation is reshuffled, are key evolutionary processes shaping genomic diversity. Importantly, these rates vary not just across populations and species but also across individual genomes. Despite previous studies having demonstrated that failing to account for rate heterogeneity across the genome can bias the inference of both selective and neutral population genetic processes, mutation and recombination rate maps have to date only been generated for a relatively small number of organisms. Here, we infer such fine‐scale maps for the aye‐aye ( *Daubentonia madagascariensis* )—a highly endangered strepsirrhine that represents one of the earliest splits in the primate clade and thus stands as an important outgroup to the more commonly studied haplorrhines—utilizing a recently released fully annotated genome combined with high‐quality population sequencing data. We compare our indirectly inferred rates to previous pedigree‐based estimates, finding further evidence of relatively low mutation and recombination rates in aye‐ayes compared to other primates.

## Graphical abstract

Despite previous studies having demonstrated that failing to account for mutation and recombination rate heterogeneity across the genome can bias the inference of both selective and neutral population genetic processes, mutation and recombination rate maps have only been generated for a relatively small number of organisms. Here we generate such maps for the aye‐aye ( *Daubentonia madagascariensis* )—a highly endangered strepsirrhine that represents one of the earliest splits in the primate clade—utilizing a newly available genome annotation combined with high‐quality, whole‐genome population sequencing data. We compare our indirectly inferred rates to previous pedigree‐based estimates, finding further evidence of relatively low mutation and recombination rates in aye‐ayes compared to other primates.

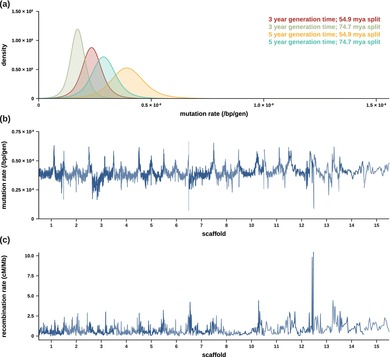

## 1 Introduction

The rate of input of new genetic variation, and the rate at which that variation is shuffled into potentially novel combinations via crossover and non‐crossover events, are fundamental evolutionary forces shaping observed genomic diversity. Over the past decades, it has become clear that mutation rates vary at a variety of scales, from between sites in a genome, to between individuals in a population, to between populations of a species, as well as broadly across the Tree of Life (see the reviews of Baer et al. 2007; Lynch 2010; Hodgkinson and Eyre‐Walker 2011; Pfeifer 2020b). The same is true of recombination, with modifications of underlying rates observed to occur at even more rapid timescales (see the reviews of Ritz et al. 2017; Stapley et al. 2017). Importantly, heterogeneity in both mutation and recombination rates across a genome can significantly alter interactions between other evolutionary processes; for example, modifying Hill‐Robertson effects (Hill and Robertson 1966; Felsenstein 1974), thereby modulating the genomic impact of selection at linked sites (Maynard Smith and Haigh 1974; Begun and Aquadro 1992; Charlesworth et al. 1993; and see Charlesworth and Jensen 2021, 2022). Furthermore, neglecting this underlying rate heterogeneity in favor of using single, species‐averaged rates for mutation and recombination—as is common practice in evolutionary models—has been shown to result in potentially misleading inference when performing downstream analyses that rely on these estimates (e.g., for inferring both population history and distributions of fitness effects, Soni et al. 2024; Soni and Jensen 2025; Soni, Versoza, Pfeifer, et al. 2025; and see Dapper and Payseur 2018; Samuk and Noor 2022; Ghafoor et al. 2023).

Aside from classical disease‐incidence approaches (e.g., Haldane 1932, 1935), there are generally two classes of experiments to infer mutation rates in primates and other large organisms. Direct mutation rate estimation relies on high‐throughput genome sequencing of parent‐offspring trios or multi‐generation pedigrees, counting the number of de novo mutations occurring from one generation to the next (see the review of Pfeifer 2020b). As mutations are rare, this generally results in only a genome‐wide estimate over the limited number of generations considered, rather than providing a finer‐scale map. Relatedly, tremendous caution must be exercised in the applied computational approach as errors introduced during sequencing will generally far outnumber genuine spontaneous mutations (Pfeifer 2021; Bergeron et al. 2022). Alternatively, indirect mutation rate estimation from species‐level divergence data instead relies on Kimura's (1968) observation that the neutral mutation rate is equal to the neutral divergence rate. Specifically, the number of substitutions *K* that accumulate in a lineage in time *T* is equal to (*μ*/*G*)*T*, where *μ* is the per‐generation mutation rate and *G* the generation time. As such, historically averaged mutation rates can be inferred from phylogenetic sequence data in neutral genomic regions, with the caveat that such estimates must generally be couched within the context of underlying uncertainties in both divergence and generation times (thus generally resulting in a range of possible mutation rates). Complicating matters further, the identification of neutral regions necessary for this indirect rate estimation requires high‐quality genome annotations that are not yet widely available for many organisms.

Similarly for recombination, taking a pedigree‐based approach enables the detection of contemporary crossover and non‐crossover events in males and females separately. As with direct mutation rate estimation, these approaches have the advantage of direct observation, though the genome‐scale resolution is again relatively coarse given the small number of meiotic exchanges that can be observed within a pedigree (see the review of Clark et al. 2010). By contrast, population‐based approaches using unrelated individuals can indirectly infer historical recombination rates from patterns of linkage disequilibrium (LD) observed in the sample (see the reviews of Stumpf and McVean 2003; Peñalba and Wolf 2020). As such, these approaches offer a higher genomic resolution and may thus provide for fine‐scale mapping, though inferred rates are necessarily sex‐averaged and may be confounded by other population‐level factors that can alter levels of LD (e.g., population history or selective effects; Dapper and Payseur 2018; Samuk and Noor 2022). For this reason, it is important to both directly model a fit demographic history when performing such inference and to carefully annotate neutral genomic regions prior to analysis (Johri et al. 2020, 2022).

In primates, many of the highest quality estimates of both mutation and recombination rates have been obtained in humans and their closest relatives (i.e., non‐human great apes) as well as in species of biomedical relevance (e.g., Kong et al. 2002; Auton et al. 2012; Stevison et al. 2016; Pfeifer 2020a; Xue et al. 2020; Wall et al. 2022; Versoza et al. 2024; Soni, Versoza, Jensen, et al. 2025). In humans, for example, large‐scale sequencing of pedigrees has yielded mutation rate estimates of ~10^−8^ per base pair per generation (see the review of Ségurel et al. 2014), which is roughly two‐fold lower than the initial indirect estimates obtained from phylogenetic data (Nachman and Crowell 2000; Kondrashov 2003); while crossover rates have been inferred to range from 0.96 to 2.11 cM/Mb for the longest and shortest autosomes, respectively, with an overall sex‐averaged rate of ~1 cM/Mb (Kong et al. 2002). Recently, however, owing to the generation of high‐quality population genomic data from pedigreed individuals, combined with the release of a fully annotated, chromosomal‐level genome assembly (Versoza and Pfeifer 2024), we now additionally have direct mutation and recombination rate estimates for aye‐ayes ( *Daubentonia madagascariensis* ), a highly endangered strepsirrhine that represents one of the earliest splits in the primate clade (Versoza, Ehmke, et al. 2025; Versoza, Jensen, and Pfeifer 2025; Versoza, Lloret‐Villas, et al. 2025). These direct estimates suggested an average genome‐wide mutation rate of ~1.1 × 10^−8^ per base pair per generation for the species—although mutation rates in the wild may be closer to a rate of ~0.4 × 10^−8^ per base pair per generation, as was estimated for individuals in the pedigree reproducing at an early age—and a sex‐averaged crossover rate of 0.85 cM/Mb. Importantly, utilizing polymorphism data from unrelated individuals, Terbot, Soni, Versoza, Pfeifer, et al. (2025) additionally estimated a well‐fitting population history for aye‐ayes (and see Soni, Terbot, Versoza, et al. 2025), describing a severe and relatively ancient population decline in the species coinciding with the arrival of humans to Madagascar, as well as a far more recent decline likely associated with habitat destruction and fragmentation over the past few decades.

Taking advantage of this newly available high‐coverage genome‐wide polymorphism data from both unrelated and pedigreed individuals, the recent annotation of the genome enabling the masking of functional (i.e., directly selected) regions, as well as these pedigree‐based direct coarse‐scale estimates allowing for meaningful comparison, we here infer indirect fine‐scale mutation and recombination rate maps across the aye‐aye genome utilizing both levels and patterns of variation as well as divergence from other closely related primate species. Aside from the biological insight into the rates of mutation and recombination gained in this study, by allowing for the incorporation of the observed rate heterogeneity, these newly developed fine‐scale maps will thus also be vitally important to improve future primate evolutionary models.

## 2 Results and Discussion

### 2.1 Fine‐Scale Mutation Rate Map

**FIGURE 1:**
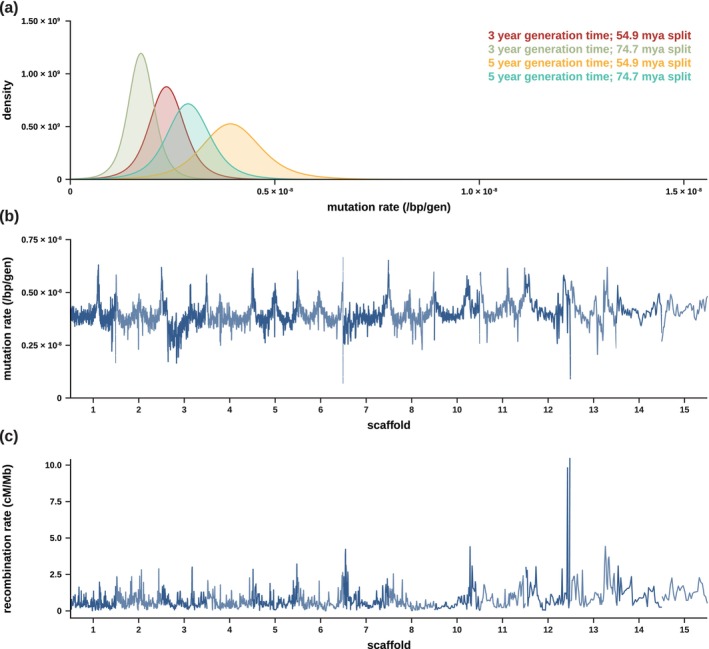
(a) Density plots of the per base pair per generation (/bp/gen) mutation rate implied by neutral divergence for two possible generation times (3 and 5 years; Ross 2003; Louis et al. 2020) and two possible divergence times (54.9 million years ago [mya] and 74.7 mya; Horvath et al. 2008). (b) Genome‐wide mutation rates for windows of size 1 Mb, with a 500 kb step size (and see Figure S2 for mutation rate heterogeneity across each individual autosomal scaffold). (c) Genome‐wide recombination rates for windows of size 1 Mb, with a 500 kb step size (and see Figure S5 for recombination rate heterogeneity across each individual autosomal scaffold).

We calculated divergence by removing the existing (but outdated) aye‐aye genome from the 447‐way multiple species alignment, consisting of the combined mammalian multiple species alignment of the Zoonomia Consortium (2020) and the primate multiple species alignment of Kuderna et al. (2024), and replaced it with the current NCBI reference genome for the species (i.e., the high‐quality, fully annotated aye‐aye genome of Versoza and Pfeifer (2024); see Section 4 for details). By masking both functional regions and segregating variants, we calculated neutral divergence across accessible sites for a range of window sizes (1, 10, 100 kb, and 1 Mb), yielding a mean neutral divergence rate of 0.043 at the 1 Mb scale relative to the reconstructed ancestor (Figure S1). Utilizing lower and upper bounds of aye‐aye divergence times (54.9 million years ago [mya] and 74.7 mya; Horvath et al. 2008) and bounds of likely generation times (3 and 5 years; Ross 2003; Louis et al. 2020), we calculated neutral mutation rates across these genomic windows, as depicted in Table 1. The average mutation rate varied from 1.73 × 10^−9^ mutations per base pair per generation (under a divergence time of 74.7 mya and a generation time of 3 years) to 3.93 × 10^−9^ mutations per base pair per generation (under a divergence time of 54.9 mya and a generation time of 5 years). Figure 1a provides density plots of mutation rates for these divergence and generation times and Figure 1b displays the heterogeneity in mutation rates across the genome (and see Figure S2 for the mutation rate heterogeneity across each individual autosomal scaffold).

**TABLE 1:** Inferred aye‐aye divergence times based on the observed mean neutral divergence rate of 0.043 for two different possible generation times (3 and 5 years; Ross 2003; Louis et al. 2020) and three different pedigree‐based mutation rates estimated for parents of differing ages by Versoza, Ehmke, et al. (2025) (shown in blue). Relatedly, the resulting divergence‐based mutation rate estimates based on two possible divergence times (54.9 million years ago [mya] and 74.7 mya; Horvath et al. 2008) and two possible generation times (3 and 5 years; Ross 2003; Louis et al. 2020) are given for comparison (shown in orange).

		Pedigree‐based mutation rate			Divergence time	
		4.0E‐09	1.1E‐08	2.0E‐08	54.9 mya	74.7 mya
Generation time (years)	3	32.3 mya	11.7 mya	6.45 mya	2.36E‐09	1.73E‐09
	5	53.8 mya	19.5 mya	10.8 mya	3.93E‐09	2.89E‐09

Taking the reverse tack, we additionally estimated aye‐aye divergence times utilizing the recently inferred mutation rates from multi‐generation aye‐aye pedigree data (Table 1; Versoza, Ehmke, et al. 2025). These rates ranged from 0.4 × 10^−8^ mutations per base pair per generation in individuals born to younger parents (< 12 years of age) to 2.0 × 10^−8^ mutations per base pair per generation in individuals born to older parents (> 24 years of age) in the pedigree, with an average rate of ~1.1 × 10^−8^ mutations per base pair per generation, resulting in estimated divergence times spanning the very large range from 53.8 to 6.45 mya (when considering the highest and lowest generation times as well). These results suggest that average ages of reproduction in the wild are comparatively young, given that the rates associated with older parents in captivity provide unrealistically recent divergence times relative to the fossil record (Gingerich 2006; Smith et al. 2006; and see the review of Gingerich 2012)—an observation in agreement with previous ecological studies that reported average reproductive ages of 3–5 years in the wild (Ross 2003; Louis et al. 2020). That said, if mean generation times were indeed considerably longer in reality, the higher mutation rate observed in older parents would help offset the disparity generated by the correspondingly fewer generations since the split of the aye‐aye branch. However, taken together, and given that the times associated with younger parents are also consistent with previous estimates of divergence based on a limited set of genetic markers encompassing ~9 kb of nuclear sequence (Horvath et al. 2008), the lower direct pedigree mutation rate of 0.4 × 10^−8^ per base pair per generation is likely the more appropriate long‐term estimate for the species (thereby also supporting a generation time estimate of ~5 years).

Given that this direct mutation rate estimate falls within our indirectly inferred mean mutation rate in this study as well, and that prosimians have been shown to have generally lower mutation rates compared to other primates (Tran and Pfeifer 2018; Chintalapati and Moorjani 2020), these results represent an accumulating body of evidence in support of relatively low mutation rates in aye‐ayes. Importantly, there is a considerable discordance in divergence time estimates of the strepsirrhine–haplorrhine split between those based on molecular data and the sparse fossil record—with the former placing the split as early as 90 mya and the latter at 55 mya (Hartwig 2011). Hence, with our improved inference of mutation rates from both pedigree‐based and divergence data, our estimate of ~53.8 mya is in agreement with the origin of primates (Tavaré et al. 2002; Zhang et al. 2008), and thus with strepsirrhines representing one of the earliest splits in the primate clade (Pozzi et al. 2014).

### 2.2 Fine‐Scale Recombination Rate Map

We inferred fine‐scale rates of recombination using pyrho (Spence and Song 2019), a re‐implementation of the software package LDhat (McVean et al. 2002, 2004; Auton and McVean 2007) employed in earlier studies investigating the landscape of recombination in non‐human primates (e.g., Auton et al. 2012; Stevison et al. 2016; Pfeifer 2020a) but that, unlike the original approach, can explicitly account for the population size change history when performing inference. To assess the performance of pyrho, we first simulated a region of 1.6 Mb (i.e., the longest accessible intergenic stretch in the aye‐aye genome) based on a fixed recombination rate (0.85 cM/Mb; Versoza, Lloret‐Villas, et al. 2025) and mutation rate (0.4 × 10^−8^ and 1.1 × 10^−8^ per base pair per generation; Versoza, Ehmke, et al. 2025), under two different demographic models, sampling five individuals to match our empirical data. The first model is the recently estimated demographic history for the species consisting of multiple population declines (Terbot, Soni, Versoza, Pfeifer, et al. 2025). Specifically, in the bottleneck‐decline model of Terbot, Soni, Versoza, Pfeifer, et al. (2025), an ancestral population size of 11,695 individuals experienced a bottleneck 1133 generations in the past, followed by a further decline at a constant rate, resulting in a population of 1285 individuals. For comparison, the second model represents a constant equilibrium model with a population size of 11,695 individuals (i.e., the inferred ancestral population size). Our simulations demonstrate that pyrho consistently underestimates recombination rates across all parameter combinations (Figure S3), despite utilizing the defined demographic model during inference. This observation is in broad agreement with a recent study examining the performance of recombination rate estimators under a variety of increasingly complex scenarios, showing that pyrho generally underestimates the population‐scaled recombination rate in declining populations if the sample size is small (see figure 1 in Dutheil 2024). These results once again emphasize the importance of evaluating the performance of statistical inference approaches within the context of the specific population and species in question for any given analysis (see Johri et al. 2022).

With these simulation‐based benchmarks on hand, we then estimated recombination rates from patterns of LD observed in the empirical data, taking into account the population size change history previously inferred by Terbot, Soni, Versoza, Pfeifer, et al. (2025). Thereby, pyrho utilizes a user‐specified, species‐specific mutation rate—here 0.4 × 10^−8^ per base pair per generation as estimated by Versoza, Ehmke, et al. (2025) for individuals reproducing at a young age, as likely the case in the wild (Ross 2003)—to convert the internally estimated population‐scaled recombination rate *ρ* to a per‐generation recombination rate *r*. Given the uncertainty in the historical effective population size (*N*_e_) of the species required for this conversion and given the underestimation in rate observed during our performance benchmarks of pyrho, we rescaled the recombination rate estimates in a way such that the total genetic map length estimated by pyrho was equal to the pedigree‐based genetic map length recently inferred by Versoza, Lloret‐Villas, et al. (2025) while retaining the relative rates across the genome (see Section 4 for details). After this rescaling, the average genome‐wide recombination rate was 0.68 cM/Mb at the 1 Mb‐scale (Figure S4)—lower than the average rate reported for anthropoid apes (~10^−8^ recombination events per base pair per generation, or ~1 cM/Mb for humans and ~1.2 cM/Mb for bonobos, chimpanzees, and gorillas; Kong et al. 2002; Auton et al. 2012; Stevison et al. 2016). However, despite the reduction in overall rate, aye‐ayes exhibit a landscape of recombination similar to those of other primates (Auton et al. 2012; Stevison et al. 2016; Pfeifer 2020a; Wall et al. 2022; Versoza et al. 2024); for example, recombination rates are generally elevated towards the telomeric ends and depressed within centromeric and pericentromeric regions of each autosomal scaffold (see Figure 1c for genome‐wide recombination rates and Figure S5 for the fine‐scale variation in recombination rates across each individual autosomal scaffold).

### 2.3 Correlations Between Fine‐Scale Rates of Recombination With Genomic Features

In order to gain a better understanding of the evolution of the recombination landscape in aye‐ayes, we studied the impact of several genomic features on scale‐specific recombination rates. To this end, we calculated nucleotide diversity and divergence based on the aye‐aye population genomic data and the 447‐way mammalian multiple species alignment as noted above, as well as GC‐content (as a measure for genome composition) and exon‐content (as a proxy for evolutionary constraint) based on the annotated aye‐aye assembly in 1 kb‐regions along the genome. We applied a discrete wavelet transformation in order to obtain information on the heterogeneity in each feature, with detail coefficients providing scale‐specific information at a range of (2^*n*^) scales. After transformation, we performed a linear model analysis of these detail coefficients to study the scale‐specific relationships between the heterogeneity in each genomic feature and recombination rate.

**FIGURE 2:**
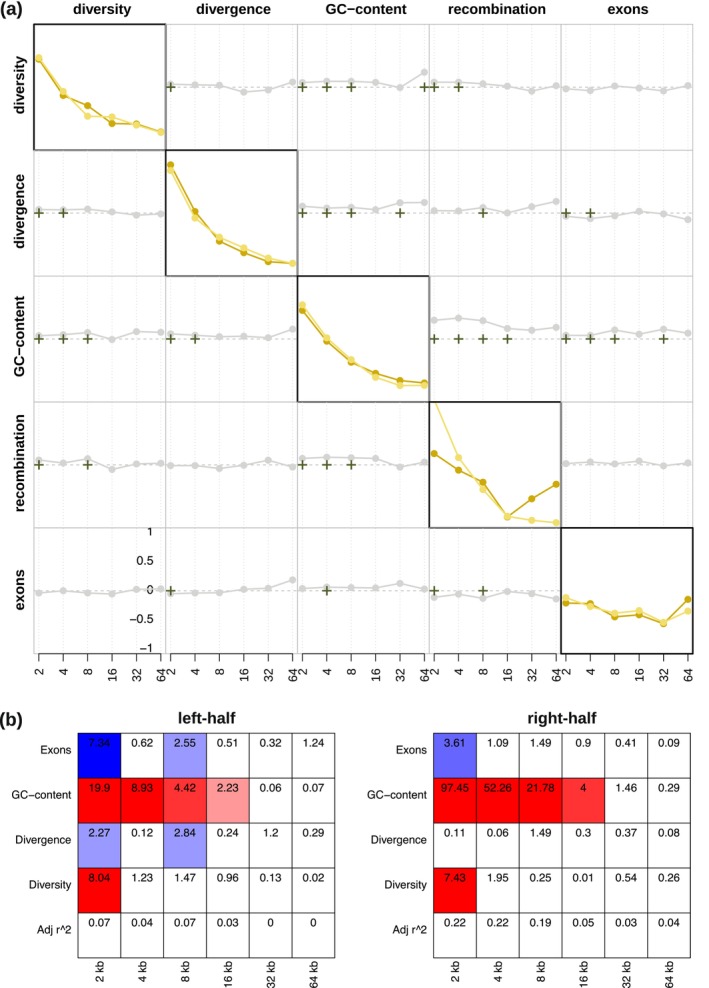
(a) The detail coefficients of each genomic feature (diagonal plots) on the left and right halves of one of the autosomal scaffolds as an illustrative example (shown in dark and light yellow, respectively) as well as their pairwise correlations based on Kendall's rank correlation (off‐diagonal plots with the bottom left showing the left‐half and the top right showing the right‐half of the scaffold) at a range of (2^*n*^) scales. Correlations significant at the 1%‐level under a two‐tailed test are highlighted by crosses. (b) Linear model analysis of the detail coefficients. Red and blue coloring indicate significant positive and negative relationships under a two‐sided *t*‐test, with the color intensity being proportional to the significance level. Adjusted *r* ^2^ specifies the proportion of heterogeneity that can be explained by the linear model. All individual autosomal scaffolds are shown in Figures S6–S19.

Figure 2a provides the detail coefficients for each genomic feature (diagonal plots) as well as their pairwise correlations (off‐diagonal plots) at scales ranging from 2^1^ to 2^12^, and Figure 2b the corresponding linear model analysis of the detail coefficients for one of the scaffolds as an illustrative example (for all autosomal scaffolds, see Figures S6–S19; for the X‐chromosome, see Terbot, Soni, Versoza, Milhaven, et al. 2025). Similar to haplorrhines (Spencer et al. 2006; Pfeifer 2020a), aye‐ayes exhibit the highest level of heterogeneity in nucleotide diversity and neutral divergence at the finest (2 kb) scale; similarly, the largest heterogeneity in recombination rate occurs over scales of 2–8 kb, the same range previously observed in monkeys (2 kb; Pfeifer 2020a) and humans (8 kb; Spencer et al. 2006). Focusing on the pairwise correlations between the detail coefficients at the fine (2–8 kb) scale, nucleotide diversity is significantly positively correlated with both neutral divergence and GC‐content, as expected given that the rate of mutation, which jointly impacts diversity and divergence, varies depending on the local base composition in the genome (Figure 1b, and see the review of Hodgkinson and Eyre‐Walker 2011). The rates of divergence are also significantly negatively correlated with exon‐content at the fine (2 kb) scale, as anticipated from evolutionary constraint to maintain proper gene function, thereby subjecting these regions to purifying selection (see the reviews of Charlesworth and Jensen 2021, 2022). In addition to mutation, and similar to other primates (Spencer et al. 2006; Auton et al. 2012; Pfeifer and Jensen 2016; Stevison et al. 2016), GC‐rich genomic regions are generally associated with higher rates of recombination in aye‐ayes. Contributing to this positive correlation at the fine scale is GC‐biased gene conversion, an evolutionary process associated with meiotic recombination that elevates the GC‐content of a region through the preferential transmission of GC over AT alleles (Duret and Galtier 2009), thus leading to a higher GC‐content in regions of frequent recombination (i.e., recombination hotspots). Additionally, in regions of high recombination, the effects of selection at linked sites (e.g., background selection and selective sweeps) will be reduced, allowing more genetic diversity to persist in close proximity (Maynard Smith and Haigh 1974; Begun and Aquadro 1992; Charlesworth et al. 1993). However, recombination hotspots are highly localized (within 1–2 kb regions; Baudat et al. 2010; Myers et al. 2010; Parvanov et al. 2010) and often flanked by regions of low recombination which, in turn, extend genetic hitchhiking effects, reducing nucleotide diversity at intermediate scales. While the small sample size and low SNP density of the dataset on hand do not allow for a detailed characterization of the hotspot landscape in aye‐ayes, higher recombination rates were observed in regions harboring the putative PRDM9 binding motif recently predicted in silico by Versoza, Lloret‐Villas, et al. (2025) (average recombination rates: 0.92 cM/Mb vs. 0.75 cM/Mb; *t* = 27.448, df = 116,494, *p*‐value < 2.2e‐16).

## 3 Concluding Thoughts

In this study, we have characterized the underlying heterogeneity in mutation and recombination rates across the genome of aye‐ayes. We found that mutation rates in this species are lower than in other primates, which is in agreement with previous studies showing lower mutation rates in prosimians (Tran and Pfeifer 2018; Chintalapati and Moorjani 2020). Notably, this indirect divergence‐based estimate supports the recent pedigree‐based estimate of 0.4 × 10^−8^ per base pair per generation characteristic of younger parents (Versoza, Ehmke, et al. 2025), suggesting a relatively young long‐term reproductive age in the wild, as might be expected from previous studies of the life history and socioecology of the species (Ross 2003). This rate also implies a split time of ~54 mya, consistent with the earliest primates in the fossil record, as opposed to the much older and difficult‐to‐reconcile split times previously proposed. Importantly, while the previously published pedigree‐based approach provided a high‐quality snapshot of contemporary rates in a single generation, because de novo mutations are rare in primates, this analysis did not provide information on mutation rate variation across the genome itself. However, by considering longer evolutionary timespans as captured from neutral divergence data, the analyses presented here have provided the first high‐quality, fine‐scale rate map across the aye‐aye genome, thereby uniquely describing comparatively high and low mutation rate regions. It is nonetheless reassuring to observe a correspondence between the single genome‐wide averaged rate as provided by direct estimation and the mean of the fine‐scale rate variation described here via indirect estimation.

We similarly found a notable reduction of recombination rate in aye‐ayes compared to the great apes (Auton et al. 2012; Stevison et al. 2016), despite overall similarities in the recombination landscape and its correlation with genomic features. Given the recently reported enrichment of crossover events in regions harboring a predicted binding motif for PRDM9 (a zinc‐finger protein controlling the activation of hotspots in primates) in pedigreed aye‐aye individuals (Versoza, Lloret‐Villas, et al. 2025), together with our observation of higher recombination rates in these regions based on polymorphism data, the future characterization of the hotspot landscape in this species should thus be of great interest to the comparative primate genomics community. Furthermore, as with mutation rates, this polymorphism‐based indirect approach has provided the first fine‐scale map of recombination rate variation across the aye‐aye genome, as compared to the single genome‐wide averaged rate provided by previous pedigree‐based analyses.

With rate maps available in only a limited number of species, it is common practice to use a single, species‐averaged rate for both mutation and recombination when modeling population genetic processes. However, failing to account for the underlying heterogeneity in mutation and recombination rates has been shown to potentially bias the inference of both population history as well as the distribution of fitness effects (e.g., Soni et al. 2023, 2024). Thus, the rate maps provided here will facilitate more robust inference of population genetic processes in the highly endangered aye‐aye specifically, as well as in evolutionary models of primate evolution more broadly.

## 4 Materials and Methods

### 4.1 Population Genomic Data

To infer fine‐scale rates of mutation and recombination, we took advantage of whole‐genome sequencing data from five unrelated aye‐aye ( *D. madagascariensis* ) individuals sequenced on an Illumina NovaSeq to a genome‐wide average coverage of > 50× per individual (Terbot, Soni, Versoza, Pfeifer, et al. 2025) to identify variants segregating in the population. Following standard quality control practices (Pfeifer 2017), we removed sequence adapters and trimmed low‐quality ends with TrimGalore v.0.6.10 (https://github.com/FelixKrueger/TrimGalore) prior to mapping the reads to the species' reference genome (DMad_hybrid; Versoza and Pfeifer 2024) with BWA‐MEM v.0.7.17 (Li and Durbin 2009). Afterward, we called, jointly genotyped, and filtered variants following the Genome Analysis Toolkit (GATK v.4.2.6.1) recommendations for germline variant discovery (van der Auwera and O'Connor 2020), adjusting parameters as needed to reflect our study design. Specifically, we set (1) the “‐‐pcr‐indel‐model” parameter to *NONE* as a PCR‐free library protocol was followed during sequencing, (2) the “‐‐heterozygosity” parameter to 0.0005 to reflect the species' genetic diversity (Perry et al. 2013), and (3) the “‐ERC” parameter to *BP_RESOLUTION* to obtain genotype information at all sites (i.e., variant as well as invariant). Supplementing GATK's recommended pipeline, we implemented a set of stringent filter criteria to eliminate spurious single nucleotide polymorphisms (SNPs) that may lead to artificial breaks in patterns of LD. Specifically, following the guidelines described in earlier studies investigating the landscape of recombination in non‐human primates (Auton et al. 2012; Stevison et al. 2016; Pfeifer 2020a), we removed both SNP clusters—defined here as three or more SNPs within a 10 bp window (calculated using the GATK v.4.2.6.1 *VariantFiltration* function together with the parameters “‐‐cluster‐size 3” and “‐‐cluster‐window‐size 10”)—as well as SNPs exhibiting an excess of heterozygosity—defined here as sites with a Hardy–Weinberg equilibrium *p*‐value of < 0.01 (calculated using the “‐‐hardy” option in VCFtools v.0.1.14; Danecek et al. 2011). Additionally, we excluded all SNPs located within regions blacklisted by the ENCODE Project Consortium (2012) (i.e., within regions prone to artifacts in high‐throughput sequencing experiments) by lifting the data between the aye‐aye (DMad_hybrid) genome assembly and the human (hg38) genome assembly using the UCSC liftOver tool (Raney et al. 2024). The resulting high‐quality, population‐level dataset consisted of 3,454,304 autosomal biallelic SNPs (transition‐transversion ratio: 2.53) in the accessible genome (Table S1).

### 4.2 Updating the Aye‐Aye Genome in the 447‐Way Mammalian Multiple Species Alignment

We obtained the 447‐way multiple species alignment, consisting of the combined mammalian multiple species alignment of the Zoonomia Consortium (2020) and the primate multiple species alignment of Kuderna et al. (2024), from https://cglgenomics.ucsc.edu/november‐2023‐nature‐zoonomia‐with‐expanded‐primates‐alignment/ and removed the outdated aye‐aye genome assembly using the *halRemoveGenome* function implemented in HAL v.2.2 (Hickey et al. 2013). Next, we added the current NCBI reference genome for the species—that is, the high‐quality, fully annotated aye‐aye assembly of Versoza and Pfeifer (2024) (DMad_hybrid; GenBank accession number: GCA_044048945.1)—to the alignment, by first extracting the ancestral genomes PrimatesAnc005 and PrimatesAnc011 from the 447‐way alignment using HAL's *hal2fasta* function, and then aligning these ancestral genomes with the new aye‐aye genome in Cactus v.2.9.2 (Armstrong et al. 2020) using the branch lengths previously inferred in the 447‐way alignment. Finally, we attached this alignment back into the 447‐way alignment using HAL's *halReplaceGenome* function.

### 4.3 Inferring Fine‐Scale Rates of Neutral Divergence and Mutation

To infer fine‐scale rates of neutral divergence and mutation, we first used the *halSummarizeMutations* function implemented in HAL v.2.2 (Hickey et al. 2013) to retrieve “point mutations” along the aye‐aye branch (i.e., substitutions between the aye‐aye and PrimateAnc005, a group consisting of several haplorrhines), and masked any sites within 10 kb of functional regions to avoid the potentially confounding effects of selection. From these point mutations, we then removed all sites associated with segregating polymorphisms in the species (Terbot, Soni, Versoza, Pfeifer, et al. 2025), resulting in a final dataset from which we calculated neutral divergence by dividing the number of divergent sites by the number of accessible sites in any given genomic window (Soni, Terbot, Versoza, et al. 2025). Specifically, divergence was estimated genome‐wide, as well as in windows of size 1, 10, 100 kb, and 1 Mb using a sliding window approach with a step size of 1, 5, 50, and 500 kb, respectively. To obtain mutation rates for each genomic window, we divided by the divergence time in generations, using divergence times of 54.9 and 74.7 mya (Horvath et al. 2008) and generation times of 3 and 5 years (Ross 2003; Louis et al. 2020) for comparison.

### 4.4 Inferring Fine‐Scale Rates of Recombination

We utilized the demography‐aware estimator pyrho v.0.1.7 (Spence and Song 2019) to estimate recombination rates from patterns of LD observed in the sequencing data. To this end, we first generated a likelihood lookup table using pyrho's *make_table* function, taking into account the population size change history previously inferred by Terbot, Soni, Versoza, Pfeifer, et al. (2025) (“‐‐popsizes 2570, 2944.784, 3374.224, 3866.288, 4430.111, 5076.157, 5816.415, 6585, 23389 ‐‐epochtimes 1,2,3,4,5,6,71,133”), and then ran the *hyperparam* function with the species‐specific mutation rate estimated by Versoza, Ehmke, et al. (2025) for individuals reproducing at a young age (“‐‐mu 0.4e‐8”), as likely the case in the wild (Ross 2003), to determine the optimal parameter settings for window size and block penalty. We then used pyrho's *optimize* function with the recommended window size of 30 (“‐‐windowsize 30”) and block penalty of 45 (“‐‐blockpenalty 45”) to estimate recombination rates across the genome. Thereby, pyrho internally estimates the population recombination rate *ρ* = 4 *N*_e_ *r*, where *N*_e_ is the effective population size and *r* is the per‐generation recombination rate, and then uses a user‐specified mutation rate/*N*_e_ to convert *ρ* to *r*. Importantly though, while the relative recombination rates estimated across the genome are expected to be stable to this conversion, the actual scaling factor often needs to be further adjusted for organisms with mutation rates different from those observed in humans (see the discussion of the developers: https://github.com/popgenmethods/pyrho). To assess the estimates, we thus plotted the cumulative genetic map length of each autosomal scaffold in R v.4.2.2 (Figure S20). After visual inspection, we removed five regions that exhibited recombination rate estimates ~100‐ to 300‐fold higher than the genome‐wide average (scaffold 3: 202,577,091–202,579,537; scaffold 7: 4,045,278–4,047,880; scaffold 7: 55,184,220–55,189,875; scaffold 11: 78,064,486–78,079,259; scaffold 12: 62,800,537–63,003,926) as the extreme estimates observed in these regions are likely the result of local assembly errors. Similar to a previous study in another non‐human primate that exhibits a lower mutation rate than humans (Wall et al. 2022), we observed that the total genetic map length estimated indirectly from patterns of LD by pyrho was considerably shorter than that recently directly inferred from crossover events observed in a three‐generation pedigree (Versoza, Lloret‐Villas, et al. 2025). Given the uncertainty in *N*_e_, we thus followed the procedure outlined in Wall et al. (2022) and rescaled the recombination rate estimates in a way such that the total genetic map length estimated by pyrho was equal to the pedigree‐based genetic map length (sex‐averaged autosomal genetic map length: 1525 cM; Versoza, Lloret‐Villas, et al. 2025) by multiplying the pyrho estimates by a factor of 14.444.

### 4.5 Assessing the Performance of Pyrho Using Simulations

To assess the performance of the demography‐aware recombination rate estimator pyrho, we used msprime v.1.3.2 (Baumdicker et al. 2022) to simulate 10 replicates of a 1.6 Mb region (i.e., the longest uninterrupted accessible intergenic region in the aye‐aye genome) with multiple parameter combinations and sampling five individuals to match our empirical data. Specifically, to test the robustness of the tool with regard to the underlying demographic history, we implemented two models in our simulations. The first is the bottleneck‐decline model from Terbot, Soni, Versoza, Pfeifer, et al. (2025) in which an ancestral population size of 11,695 individuals experienced a bottleneck 1133 generations in the past, followed by a further decline at a constant rate, resulting in a population of 1285 individuals. The second is a constant equilibrium model with a population size of 11,695 individuals (i.e., the estimated ancestral population size). Moreover, in addition to the species‐specific average mutation rate recently estimated from a 14‐individual three‐generation pedigree in Versoza, Ehmke, et al. (2025) (1.1 × 10^−8^ per base pair per generation), we also considered the lowest reported pedigree estimate (0.4 × 10^−8^ per base pair per generation) in our models to account for individuals potentially reproducing at a young age in the wild. Finally, we used the coarse‐scale recombination rate estimate from pedigreed individuals (0.85 cM/Mb) reported in Versoza, Lloret‐Villas, et al. (2025) in all models.

### 4.6 Assessing the Correlation of Fine‐Scale Rates of Recombination With Genomic Features

Following previous work in humans (Spencer et al. 2006), we first calculated nucleotide diversity and divergence based on the aye‐aye population genomic data and the 447‐way mammalian multiple species alignment as noted above, as well as GC‐content (as a measure of base composition) and exon‐content (as a proxy for evolutionary constraint) based on the annotated aye‐aye (DMad_hybrid) genome assembly (GenBank accession number: GCA_044048945.1; Versoza and Pfeifer 2024) in 1 kb windows along the 14 autosomal scaffolds (i.e., scaffolds 1–8 and 10–15), taking into account the number of sites accessible to this study. We then applied a discrete (Haar) wavelet transformation using the *Rwave* and *wavethresh* packages implemented in R v.4.2.2 to obtain information on the heterogeneity in each genomic feature at varying scales. To study scale‐specific correlations, we additionally performed a linear model analysis on the log‐transformed recombination, nucleotide diversity, and divergence rates with the intercept forced through the origin.

## Author Contributions

**Vivak Soni:** formal analysis (equal), investigation (equal), methodology (equal), software (equal), validation (equal), visualization (equal), writing – original draft (equal). **Cyril J. Versoza:** formal analysis (equal), investigation (equal), methodology (equal), software (equal), validation (equal), visualization (equal), writing – original draft (equal). **John W. Terbot II:** formal analysis (equal), investigation (equal), methodology (equal), writing – original draft (equal). **Jeffrey D. Jensen:** conceptualization (equal), funding acquisition (equal), supervision (equal), writing – original draft (equal). **Susanne P. Pfeifer:** conceptualization (equal), formal analysis (equal), funding acquisition (equal), supervision (equal), writing – original draft (equal).

## Conflicts of Interest

The authors declare no conflicts of interest.

## Supporting information


**Appendix S1:** ece372314‐sup‐0001‐AppendixS1.pdf.

## Data Availability

This study was based on whole‐genome high‐throughput sequencing data available under NCBI BioProjects PRJNA1085541 and PRJNA1179987. The mutation and recombination rate maps generated in this study are available at https://github.com/vivaksoni/aye_aye_mutation_recombination_rates and http://spfeiferlab.org/data, respectively.

## References

[ece372314-bib-0001] Armstrong, J. , G. Hickey , M. Diekhans , et al. 2020. “Progressive Cactus Is a Multiple‐Genome Aligner for the Thousand‐Genome Era.” Nature 587, no. 7833: 246–251. 33177663 10.1038/s41586-020-2871-y PMC7673649

[ece372314-bib-0002] Auton, A. , A. Fledel‐Alon , S. Pfeifer , et al. 2012. “A Fine‐Scale Chimpanzee Genetic Map From Population Sequencing.” Science 336, no. 6078: 193–198. 22422862 10.1126/science.1216872 PMC3532813

[ece372314-bib-0003] Auton, A. , and G. McVean . 2007. “Recombination Rate Estimation in the Presence of Hotspots.” Genome Research 17, no. 8: 1219–1227. 17623807 10.1101/gr.6386707 PMC1933511

[ece372314-bib-0004] Baer, C. F. , M. M. Miyamoto , and D. R. Denver . 2007. “Mutation Rate Variation in Multicellular Eukaryotes: Causes and Consequences.” Nature Reviews Genetics 8, no. 8: 619–631. 10.1038/nrg2158 17637734

[ece372314-bib-0005] Baudat, F. , J. Buard , C. Grey , et al. 2010. “PRDM9 Is a Major Determinant of Meiotic Recombination Hotspots in Humans and Mice.” Science 327, no. 5967: 836–840. 20044539 10.1126/science.1183439 PMC4295902

[ece372314-bib-0006] Baumdicker, F. , G. Bisschop , D. Goldstein , et al. 2022. “Efficient Ancestry and Mutation Simulation With Msprime 1.0.” Genetics 220, no. 3: iyab229. 34897427 10.1093/genetics/iyab229 PMC9176297

[ece372314-bib-0007] Begun, D. J. , and C. F. Aquadro . 1992. “Levels of Naturally Occurring DNA Polymorphism Correlate With Recombination Rates in *D. melanogaster* .” Nature 356, no. 6369: 519–520. 1560824 10.1038/356519a0

[ece372314-bib-0008] Bergeron, L. A. , S. Besenbacher , T. N. Turner , et al. 2022. “The Mutationathon Highlights the Importance of Reaching Standardization in Estimates of Pedigree‐Based Germline Mutation Rates.” eLife 11: e73577. 35018888 10.7554/eLife.73577 PMC8830884

[ece372314-bib-0009] Charlesworth, B. , and J. D. Jensen . 2021. “Effects of Selection at Linked Sites on Patterns of Genetic Variability.” Annual Review of Ecology, Evolution, and Systematics 52: 177–197. 10.1146/annurev-ecolsys-010621-044528 PMC10120885 37089401

[ece372314-bib-0010] Charlesworth, B. , and J. D. Jensen . 2022. “How Can We Resolve Lewontin's Paradox?” Genome Biology and Evolution 14, no. 7: evac096. 35738021 10.1093/gbe/evac096 PMC9305300

[ece372314-bib-0011] Charlesworth, B. , M. T. Morgan , and D. Charlesworth . 1993. “The Effect of Deleterious Mutations on Neutral Molecular Variation.” Genetics 134, no. 4: 1289–1303. 8375663 10.1093/genetics/134.4.1289 PMC1205596

[ece372314-bib-0012] Chintalapati, M. , and P. Moorjani . 2020. “Evolution of the Mutation Rate Across Primates.” Current Opinion in Genetics & Development 62: 58–64. 32634682 10.1016/j.gde.2020.05.028

[ece372314-bib-0013] Clark, A. G. , X. Wang , and T. Matise . 2010. “Contrasting Methods of Quantifying Fine Structure of Human Recombination.” Annual Review of Genomics and Human Genetics 11: 45–64. 10.1146/annurev-genom-082908-150031 PMC2980829 20690817

[ece372314-bib-0014] Danecek, P. , A. Auton , G. Abecasis , et al. 2011. “The Variant Call Format and VCFtools.” Bioinformatics 27, no. 15: 2156–2158. 21653522 10.1093/bioinformatics/btr330 PMC3137218

[ece372314-bib-0015] Dapper, A. L. , and B. A. Payseur . 2018. “Effects of Demographic History on the Detection of Recombination Hotspots From Linkage Disequilibrium.” Molecular Biology and Evolution 35, no. 2: 335–353. 29045724 10.1093/molbev/msx272 PMC5850621

[ece372314-bib-0016] Duret, L. , and N. Galtier . 2009. “Biased Gene Conversion and the Evolution of Mammalian Genomic Landscapes.” Annual Review of Genomics and Human Genetics 10: 285–311. 10.1146/annurev-genom-082908-150001 19630562

[ece372314-bib-0017] Dutheil, J. Y. 2024. “On the Estimation of Genome‐Average Recombination Rates.” Genetics 227, no. 2: iyae051. 38565705 10.1093/genetics/iyae051 PMC11232287

[ece372314-bib-0018] ENCODE Project Consortium . 2012. “An Integrated Encyclopedia of DNA Elements in the Human Genome.” Nature 489, no. 7414: 57–74. 22955616 10.1038/nature11247 PMC3439153

[ece372314-bib-0019] Felsenstein, J. 1974. “The Evolutionary Advantage of Recombination.” Genetics 78, no. 2: 737–756. 4448362 10.1093/genetics/78.2.737 PMC1213231

[ece372314-bib-0020] Ghafoor, S. , J. Santos , C. J. Versoza , J. D. Jensen , and S. P. Pfeifer . 2023. “The Impact of Sample Size and Population History on Observed Mutational Spectra: A Case Study in Human and Chimpanzee Populations.” Genome Biology and Evolution 15, no. 3: evad019. 36790107 10.1093/gbe/evad019 PMC9989333

[ece372314-bib-0021] Gingerich, P. D. 2006. “Environment and Evolution Through the Paleocene‐Eocene Thermal Maximum.” Trends in Ecology & Evolution 21, no. 5: 246–253. 16697910 10.1016/j.tree.2006.03.006

[ece372314-bib-0022] Gingerich, P. D. 2012. “Primates in the Eocene.” Palaeobiodiversity and Palaeoenvironments 92: 649–663.

[ece372314-bib-0023] Haldane, J. B. S. 1932. The Causes of Evolution. Longmans, Green, & Co.

[ece372314-bib-0024] Haldane, J. B. S. 1935. “The Rate of Spontaneous Mutation of a Human Gene.” Journal of Genetics 31: 317–326. 10.1007/BF02717892 15689625

[ece372314-bib-0025] Hartwig, W. 2011. “Chapter 3: Primate Evolution.” In Primates in Perspective, edited by C. J. Campbell , A. Fuentes , K. MacKinnon , S. Bearder , and R. Stumpf , 2nd ed., 19–31. Oxford University Press.

[ece372314-bib-0026] Hickey, G. , B. Paten , D. Earl , D. Zerbino , and D. Haussler . 2013. “HAL: A Hierarchical Format for Storing and Analyzing Multiple Genome Alignments.” Bioinformatics 29, no. 10: 1341–1342. 23505295 10.1093/bioinformatics/btt128 PMC3654707

[ece372314-bib-0027] Hill, W. G. , and A. Robertson . 1966. “The Effect of Linkage on Limits to Artificial Selection.” Genetical Research 8, no. 3: 269–294. 5980116

[ece372314-bib-0028] Hodgkinson, A. , and A. Eyre‐Walker . 2011. “Variation in the Mutation Rate Across Mammalian Genomes.” Nature Reviews. Genetics 12, no. 11: 756–766. 10.1038/nrg3098 21969038

[ece372314-bib-0029] Horvath, J. E. , D. W. Weisrock , S. L. Embry , et al. 2008. “Development and Application of a Phylogenomic Toolkit: Resolving the Evolutionary History of Madagascar's Lemurs.” Genome Research 18, no. 3: 489–499. 18245770 10.1101/gr.7265208 PMC2259113

[ece372314-bib-0030] Jennewein, D. M. , J. Lee , C. Kurtz , et al. 2023. “The Sol Supercomputer at Arizona State University.” In Practice and Experience in Advanced Research Computing, 296–301. https://openondemand.org/sites/default/files/documents/PEARC23_ASU‐Sol‐paper.pdf.

[ece372314-bib-0031] Johri, P. , C. F. Aquadro , M. Beaumont , et al. 2022. “Recommendations for Improving Statistical Inference in Population Genomics.” PLoS Biology 20, no. 5: e3001669. 35639797 10.1371/journal.pbio.3001669 PMC9154105

[ece372314-bib-0032] Johri, P. , B. Charlesworth , and J. D. Jensen . 2020. “Toward an Evolutionarily Appropriate Null Model: Jointly Inferring Demography and Purifying Selection.” Genetics 215, no. 1: 173–192. 32152045 10.1534/genetics.119.303002 PMC7198275

[ece372314-bib-0033] Kimura, M. 1968. “Evolutionary Rate at the Molecular Level.” Nature 217, no. 5129: 624–626. 5637732 10.1038/217624a0

[ece372314-bib-0034] Kondrashov, A. S. 2003. “Direct Estimates of Human per Nucleotide Mutation Rates at 20 Loci Causing Mendelian Diseases.” Human Mutation 21, no. 1: 12–27. 12497628 10.1002/humu.10147

[ece372314-bib-0035] Kong, A. , D. F. Gudbjartsson , J. Sainz , et al. 2002. “A High‐Resolution Recombination Map of the Human Genome.” Nature Genetics 31, no. 3: 241–247. 12053178 10.1038/ng917

[ece372314-bib-0036] Kuderna, L. F. K. , J. C. Ulirsch , S. Rashid , et al. 2024. “Identification of Constrained Sequence Elements Across 239 Primate Genomes.” Nature 625, no. 7996: 735–742. 38030727 10.1038/s41586-023-06798-8 PMC10808062

[ece372314-bib-0037] Li, H. , and R. Durbin . 2009. “Fast and Accurate Short Read Alignment With Burrows–Wheeler Transform.” Bioinformatics 25, no. 14: 1754–1760. 19451168 10.1093/bioinformatics/btp324 PMC2705234

[ece372314-bib-0038] Louis, E. , T. Sefczek , D. Randimbiharinirina , et al. 2020. “*Daubentonia madagascariensis*. IUCN Red List Threat. Species.”

[ece372314-bib-0039] Lynch, M. 2010. “Evolution of the Mutation Rate.” Trends in Genetics 26, no. 8: 345–352. 20594608 10.1016/j.tig.2010.05.003 PMC2910838

[ece372314-bib-0040] Maynard Smith, J. , and J. Haigh . 1974. “The Hitch‐Hiking Effect of a Favourable Gene.” Genetic Resources and Crop Evolution 23, no. 1: 23–35. 4407212

[ece372314-bib-0041] McVean, G. , P. Awadalla , and P. Fearnhead . 2002. “A Coalescent‐Based Method for Detecting and Estimating Recombination From Gene Sequences.” Genetics 160, no. 3: 1231–1241. 11901136 10.1093/genetics/160.3.1231 PMC1462015

[ece372314-bib-0042] McVean, G. A. T. , S. R. Myers , S. Hunt , P. Deloukas , D. R. Bentley , and P. Donnelly . 2004. “The Fine‐Scale Structure of Recombination Rate Variation in the Human Genome.” Science 304, no. 5670: 581–584. 15105499 10.1126/science.1092500

[ece372314-bib-0043] Myers, S. , R. Bowden , A. Tumian , et al. 2010. “Drive Against Hotspot Motifs in Primates Implicates the PRDM9 Gene in Meiotic Recombination.” Science 327, no. 5967: 876–879. 20044541 10.1126/science.1182363 PMC3828505

[ece372314-bib-0044] Nachman, M. W. , and S. L. Crowell . 2000. “Estimate of the Mutation Rate per Nucleotide in Humans.” Genetics 156, no. 1: 297–304. 10978293 10.1093/genetics/156.1.297 PMC1461236

[ece372314-bib-0045] Parvanov, E. D. , P. M. Petkov , and K. Paigen . 2010. “PRDM9 Controls Activation of Mammalian Recombination Hotspots.” Science 327, no. 5967: 835. 20044538 10.1126/science.1181495 PMC2821451

[ece372314-bib-0046] Peñalba, J. V. , and J. B. W. Wolf . 2020. “From Molecules to Populations: Appreciating and Estimating Recombination Rate Variation.” Nature Reviews Genetics 21, no. 8: 476–492. 10.1038/s41576-020-0240-1 32472059

[ece372314-bib-0047] Perry, G. H. , E. E. Louis , A. Ratan , et al. 2013. “Aye‐Aye Population Genomic Analyses Highlight an Important Center of Endemism in Northern Madagascar.” Proceedings of the National Academy of Sciences 110, no. 15: 5823–5828. 10.1073/pnas.1211990110 PMC3625347 23530231

[ece372314-bib-0048] Pfeifer, S. P. 2017. “From Next‐Generation Resequencing Reads to a High‐Quality Variant Data Set.” Heredity (Edinb) 118, no. 2: 111–124. 27759079 10.1038/hdy.2016.102 PMC5234474

[ece372314-bib-0049] Pfeifer, S. P. 2020a. “A Fine‐Scale Genetic Map for Vervet Monkeys.” Molecular Biology and Evolution 37, no. 7: 1855–1865. 32211856 10.1093/molbev/msaa079 PMC7825483

[ece372314-bib-0050] Pfeifer, S. P. 2020b. “Spontaneous Mutation Rates.” In The Molecular Evolutionary Clock. Theory and Practice, edited by S. Y. W. Ho , 35–44. Springer Nature.

[ece372314-bib-0051] Pfeifer, S. P. 2021. “Studying Mutation Rate Evolution in Primates‐The Effects of Computational Pipelines and Parameter Choices.” Giga Science 10, no. 10: giab069. 34673929 10.1093/gigascience/giab069 PMC8529961

[ece372314-bib-0052] Pfeifer, S. P. , and J. D. Jensen . 2016. “The Impact of Linked Selection in Chimpanzees: A Comparative Study.” Genome Biology and Evolution 8, no. 10: 3202–3208. 27678122 10.1093/gbe/evw240 PMC5174744

[ece372314-bib-0053] Pordes, R. , D. Petravick , B. Kramer , et al. 2007. “The Open Science Grid.” Journal of Physics Conference Series 78: 012057.

[ece372314-bib-0054] Pozzi, L. , J. A. Hodgson , A. S. Burrell , K. N. Sterner , R. L. Raaum , and T. R. Disotell . 2014. “Primate Phylogenetic Relationships and Divergence Dates Inferred From Complete Mitochondrial Genomes.” Molecular Phylogenetics and Evolution 75: 165–183. 24583291 10.1016/j.ympev.2014.02.023 PMC4059600

[ece372314-bib-0055] Raney, B. J. , G. P. Barber , A. Benet‐Pagès , et al. 2024. “The UCSC Genome Browser Database: 2024 Update.” Nucleic Acids Research 52, no. D1: D1082–D1088. 37953330 10.1093/nar/gkad987 PMC10767968

[ece372314-bib-0056] Ritz, R. R. , M. A. F. Noor , and N. D. Singh . 2017. “Variation in Recombination Rate: Adaptive or Not?” Trends in Genetics 33, no. 5: 364–374. 28359582 10.1016/j.tig.2017.03.003

[ece372314-bib-0057] Ross, C. 2003. “Life History, Infant Care Strategies, and Brain Size in Primates.” In Primate Life Histories and Socioecology, edited by P. M. Kappeler and M. E. Pereira , 266–284. Chicago University Press.

[ece372314-bib-0058] Samuk, K. , and M. A. F. Noor . 2022. “Gene Flow Biases Population Genetic Inference of Recombination Rate.” G3 (Bethesda) 12, no. 11: jkac236. 36103705 10.1093/g3journal/jkac236 PMC9635666

[ece372314-bib-0059] Ségurel, L. , M. J. Wyman , and M. Przeworski . 2014. “Determinants of Mutation Rate Variation in the Human Germline.” Annual Review of Genomics and Human Genetics 15: 47–70. 10.1146/annurev-genom-031714-125740 25000986

[ece372314-bib-0060] Sfiligoi, I. , D. C. Bradley , B. Holzman , P. Mhashilkar , S. Padhi , and F. Würthwein . 2009. “The Pilot Way to Grid Resources Using glideinWWS.” In 2009 WRI World Congress on Computer Science and Information Engineering, 428–432. IEEE.

[ece372314-bib-0061] Smith, T. , K. D. Rose , and P. D. Gingerich . 2006. “Rapid Asia‐Europe‐North America Geographic Dispersal of Earliest Eocene Primate Teilhardina During the Paleocene‐Eocene Thermal Maximum.” Proceedings of the National Academy of Sciences of the United States of America 103, no. 30: 11223–11227. 16847264 10.1073/pnas.0511296103 PMC1544069

[ece372314-bib-0062] Soni, V. , and J. D. Jensen . 2025. “Inferring Demographic and Selective Histories From Population Genomic Data Using a Two‐Step Approach in Species With Coding‐Sparse Genomes: An Application to Human Data.” G3 (Bethesda) 15, no. 4: jkaf019. 39883523 10.1093/g3journal/jkaf019 PMC12005166

[ece372314-bib-0063] Soni, V. , P. Johri , and J. D. Jensen . 2023. “Evaluating Power to Detect Recurrent Selective Sweeps Under Increasingly Realistic Evolutionary Null Models.” Evolution 77, no. 10: 2113–2127. 37395482 10.1093/evolut/qpad120 PMC10547124

[ece372314-bib-0064] Soni, V. , S. P. Pfeifer , and J. D. Jensen . 2024. “The Effect of Mutation and Recombination Rate Heterogeneity on the Inference of Demography and the Distribution of Fitness Effects.” Genome Biology and Evolution 16, no. 2: evae004. 38207127 10.1093/gbe/evae004 PMC10834165

[ece372314-bib-0065] Soni, V. , J. W. Terbot , C. J. Versoza , S. P. Pfeifer , and J. D. Jensen . 2025. “A Whole‐Genome Scan for Evidence of Recent Positive and Balancing Selection in Aye‐Ayes ( *Daubentonia madagascariensis* ) Utilizing a Well‐Fit Evolutionary Baseline Model.” G3 (Bethesda) 15: jkaf078. 40208178 10.1093/g3journal/jkaf078 PMC12239616

[ece372314-bib-0066] Soni, V. , C. Versoza , J. D. Jensen , and S. P. Pfeifer . 2025. “Inferring the Landscape of Mutation and Recombination in the Common Marmoset ( *Callithrix jacchus* ) in the Presence of Twinning and Hematopoietic Chimerism.” *BioRxiv*, Preprint. 10.1101/2025.07.01.662565v1.full.pdf. PMC13487337 42574666

[ece372314-bib-0067] Soni, V. , C. Versoza , S. P. Pfeifer , and J. D. Jensen . 2025. “Estimating the Distribution of Fitness Effects in Aye‐Ayes ( *Daubentonia madagascariensis* ), Accounting for Population History as Well as Mutation and Recombination Rate Heterogeneity.” American Journal of Primatology 87: e70058. 40596780 10.1002/ajp.70058 PMC12643138

[ece372314-bib-0068] Spence, J. P. , and Y. S. Song . 2019. “Inference and Analysis of Population‐Specific Fine‐Scale Recombination Maps Across 26 Diverse Human Populations.” Science Advances 5, no. 10: eaaw9206. 31681842 10.1126/sciadv.aaw9206 PMC6810367

[ece372314-bib-0069] Spencer, C. C. , P. Deloukas , S. Hunt , et al. 2006. “The Influence of Recombination on Human Genetic Diversity.” PLoS Genetics 2, no. 9: e148. 17044736 10.1371/journal.pgen.0020148 PMC1575889

[ece372314-bib-0070] Stapley, J. , P. G. D. Feulner , S. E. Johnston , A. W. Santure , and C. M. Smadja . 2017. “Variation in Recombination Frequency and Distribution Across Eukaryotes: Patterns and Processes.” Philosophical Transactions of the Royal Society of London. Series B, Biological Sciences 372, no. 1736: 20160455. 29109219 10.1098/rstb.2016.0455 PMC5698618

[ece372314-bib-0071] Stevison, L. S. , A. E. Woerner , J. M. Kidd , et al. 2016. “The Time Scale of Recombination Rate Evolution in Great Apes.” Molecular Biology and Evolution 33, no. 4: 928–945. 26671457 10.1093/molbev/msv331 PMC5870646

[ece372314-bib-0072] Stumpf, M. P. H. , and G. A. T. McVean . 2003. “Estimating Recombination Rates From Population‐Genetic Data.” Nature Reviews Genetics 4, no. 12: 959–968. 10.1038/nrg1227 14631356

[ece372314-bib-0073] Tavaré, S. , C. R. Marshall , O. Will , C. Soligo , and R. D. Martin . 2002. “Using the Fossil Record to Estimate the Age of the Last Common Ancestor of Extant Primates.” Nature 416, no. 6882: 726–729. 11961552 10.1038/416726a

[ece372314-bib-0074] Terbot, J. W. , V. Soni , C. J. Versoza , et al. 2025. “Interpreting Patterns of X Chromosomal Relative to Autosomal Diversity in Aye‐Ayes ( *Daubentonia madagascariensis* ).” *BioRxiv*, preprint. 10.1101/2025a.01.25.634876v1. PMC12682215 41353595

[ece372314-bib-0075] Terbot, J. W. , V. Soni , C. J. Versoza , S. P. Pfeifer , and J. D. Jensen . 2025. “Inferring the Demographic History of Aye‐Ayes ( *Daubentonia madagascariensis* ) From High‐Quality, Whole‐Genome, Population‐Level Data.” Genome Biology and Evolution 17, no. 1: evae281. 39749927 10.1093/gbe/evae281 PMC11746965

[ece372314-bib-0076] Tran, L. A. , and S. P. Pfeifer . 2018. “Germ Line Mutation Rates in Old World Monkeys.” In eLS, edited by John Wiley & Sons, Ltd , 1st ed., 1–10. Wiley.

[ece372314-bib-0077] van der Auwera, G. , and B. O'Connor . 2020. Genomics in the Cloud. 1st ed. O'Reilly Media.

[ece372314-bib-0078] Versoza, C. , S. Weiss , R. Johal , B. La Rosa , J. D. Jensen , and S. P. Pfeifer . 2024. “Novel Insights Into the Landscape of Crossover and Non‐Crossover Events in Rhesus Macaques ( *Macaca mulatta* ).” Genome Biology and Evolution 16, no. 1: evad223. 38051960 10.1093/gbe/evad223 PMC10773715

[ece372314-bib-0079] Versoza, C. J. , E. Ehmke , J. D. Jensen , and S. P. Pfeifer . 2025. “Characterizing the Rates and Patterns of De Novo Germline Mutations in the Aye‐Aye (*Daubentonia madagascariensis*).” Molecular Biology and Evolution 42, no. 3: msaf034. 40048663 10.1093/molbev/msaf034 PMC11884812

[ece372314-bib-0080] Versoza, C. J. , J. D. Jensen , and S. P. Pfeifer . 2025. “The Landscape of Structural Variation in Aye‐Ayes ( *Daubentonia madagascariensis* ).” Genome Biology and Evolution 17: evaf167. 40879756 10.1093/gbe/evaf167 PMC12448223

[ece372314-bib-0081] Versoza, C. J. , A. Lloret‐Villas , J. D. Jensen , and S. P. Pfeifer . 2025. “A Pedigree‐Based Map of Crossovers and Non‐Crossovers in Aye‐Ayes (*Daubentonia madagascariensis*).” Genome Biology and Evolution 17: evaf072. 40242950 10.1093/gbe/evaf072 PMC12079367

[ece372314-bib-0082] Versoza, C. J. , and S. P. Pfeifer . 2024. “A Hybrid Genome Assembly of the Endangered Aye‐Aye ( *Daubentonia madagascariensis* ).” G3 (Bethesda) 14, no. 10: jkae185. 39109845 10.1093/g3journal/jkae185 PMC11457058

[ece372314-bib-0083] Wall, J. D. , J. A. Robinson , and L. A. Cox . 2022. “High‐Resolution Estimates of Crossover and Noncrossover Recombination From a Captive Baboon Colony.” Genome Biology and Evolution 14, no. 4: evac040. 35325119 10.1093/gbe/evac040 PMC9048888

[ece372314-bib-0084] Xue, C. , N. Rustagi , X. Liu , et al. 2020. “Reduced Meiotic Recombination in Rhesus Macaques and the Origin of the Human Recombination Landscape.” PLoS One 15: e0236285. 32841250 10.1371/journal.pone.0236285 PMC7447010

[ece372314-bib-0085] Zhang, R. , Y.‐Q. Wang , and B. Su . 2008. “Molecular Evolution of a Primate‐Specific microRNA Family.” Molecular Biology and Evolution 25, no. 7: 1493–1502. 18417486 10.1093/molbev/msn094

[ece372314-bib-0086] Zoonomia Consortium . 2020. “A Comparative Genomics Multitool for Scientific Discovery and Conservation.” Nature 587, no. 7833: 240–245. 33177664 10.1038/s41586-020-2876-6 PMC7759459

