# Supplementary material for: Inferring Fine‐Scale Mutation and Recombination Rate Maps in Aye‐Ayes (Daubentonia madagascariensis)
Source: Ecol Evol. 2025 Nov 3;15(11):e72314. doi: 10.1002/ece3.72314 (PMC12582854; doi:10.1002/ece3.72314)
Supplement: Supplementary file 1 — Appendix S1: ece372314‐sup‐0001‐AppendixS1.pdf. [file ECE3-15-e72314-s001.pdf]

Supplementary Table S1

scaffold	length	# SNPs	# invariant sites	Ts/Tv
1	316,165,773	479,977	305,422,802	2.46
2	290,592,686	432,448	280,458,013	2.49
3	261,424,170	414,954	141,303,800	2.42
4	219,686,500	337,550	212,387,742	2.47
5	215,448,047	322,953	208,264,453	2.54
6	204,016,426	299,217	197,496,960	2.56
7	199,604,927	306,171	191,642,522	2.52
8	162,769,830	237,994	157,237,269	2.50
10	114,896,738	165,249	110,918,925	2.60
11	102,076,017	145,330	98,878,083	2.83
12	67,301,774	103,688	64,014,911	2.80
13	62,733,483	100,090	59,642,869	2.78
14	34,254,822	59,203	32,493,188	2.73
15	28,257,198	49,480	27,454,529	2.74
$\sum$ or $\varnothing$	**2,279,228,391**	**3,454,304**	**2,087,616,066**	**2.52**

Supplementary Table S1. Summary of genomic data. After filtering, a total of 3.45 million autosomal, biallelic, single nucleotide polymorphisms (SNPs) with a transition-transversion ratio (Ts/Tv) of 2.52 were discovered in the accessible genome.

Supplementary Figure S1

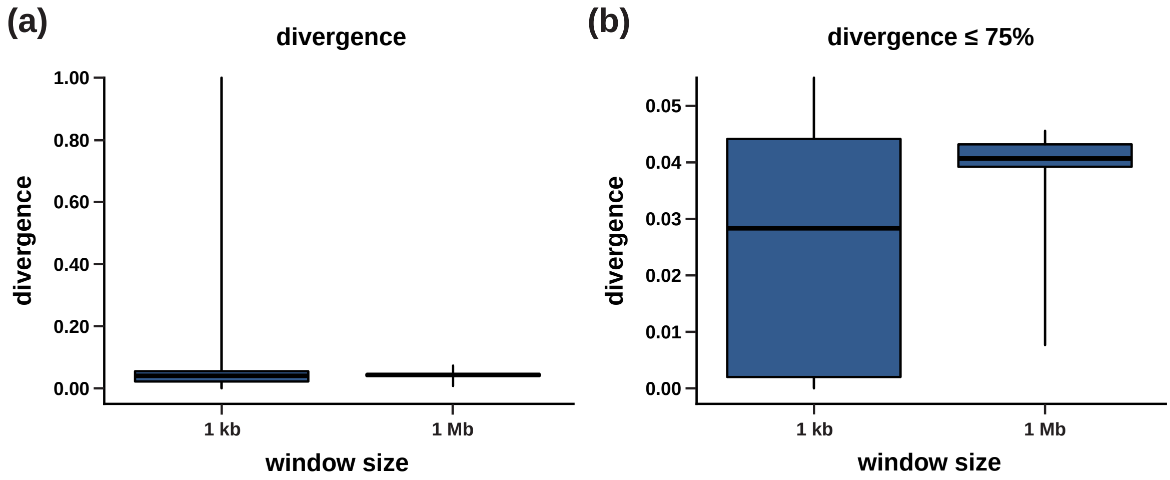

Supplementary Figure S1: Distribution of divergence for (a) all 1kb and 1Mb genomic windows and (b) for windows in the lower three quartiles. Bold bars represent median divergence, with boxes representing the 25% and 75% percentiles.

Supplementary Figure S2

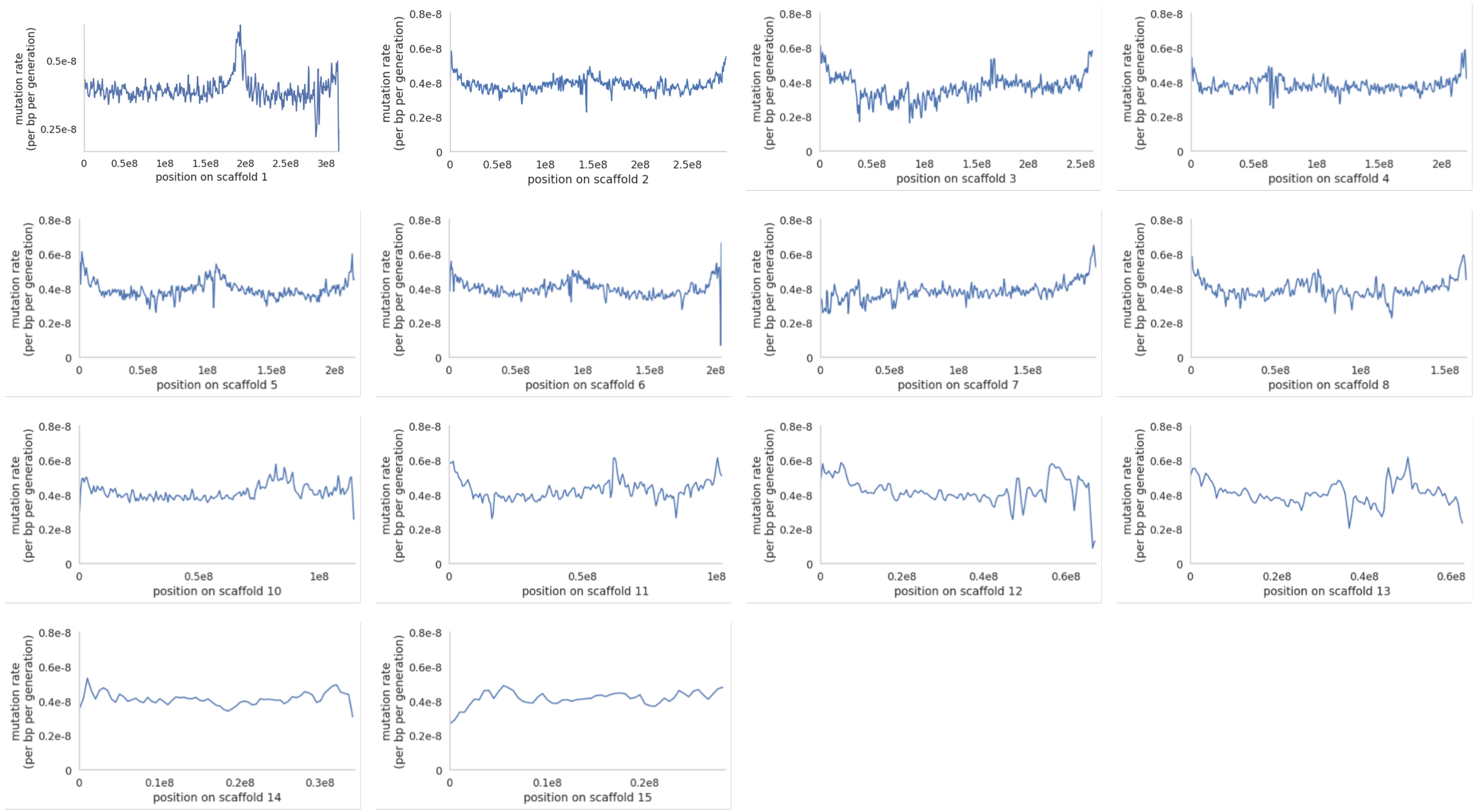

Supplementary Figure S2: Fine-scale mutation rates along each autosomal scaffold for genomic windows of size 1Mb, with a 500kb step size.

Supplementary Figure S3

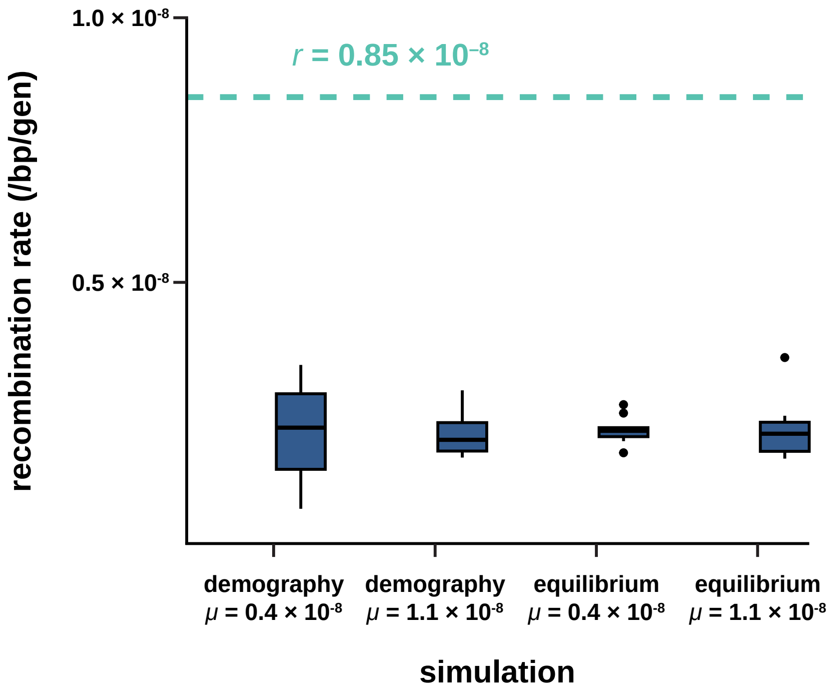

Supplementary Figure S3: Performance of the demography-aware recombination rate estimator pyrho across varying mutation rates (μ = 0.4 x 10^{-8} and 1.1 x 10^{-8} per base pair per generation [/bp/gen]; Versoza et al. 2025a) and demographic histories, including the demographic history recently estimated by Terbot et al. (2025) for the species consisting of multiple population declines (demography) as well as a constant population size (equilibrium) for comparison. The teal dashed line depicts the recombination rate that was used in the simulations (i.e., 0.85 cM/Mb; Versoza, Lloret-Villas et al. 2025).

Supplementary Figure S4

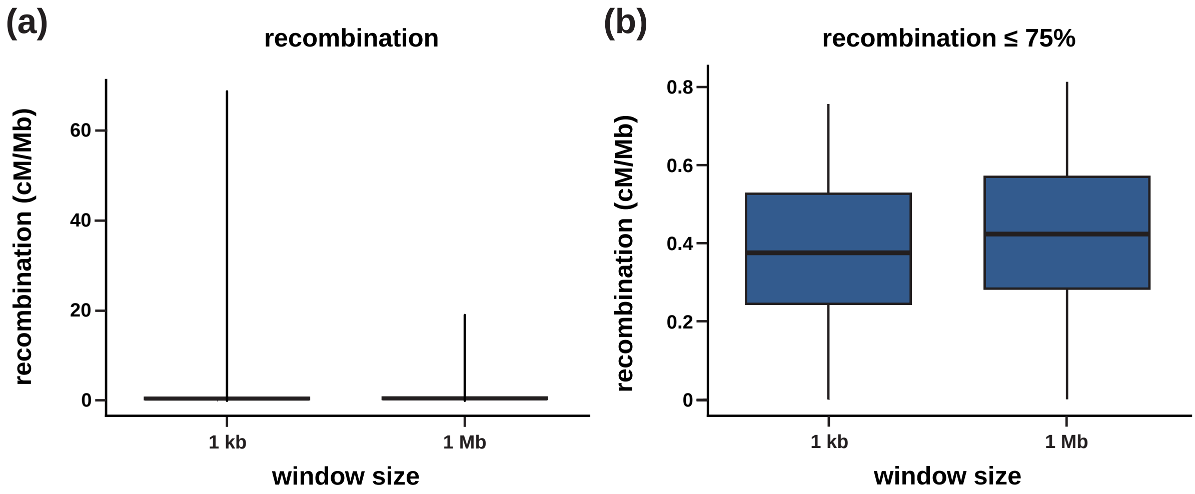

Supplementary Figure S4: Distribution of per-generation recombination rates for (a) all 1kb and 1Mb genomic windows and (b) for windows in the lower three quartiles. Bold bars represent median recombination rates, with boxes representing the 25% and 75% percentiles.

Supplementary Figure S5

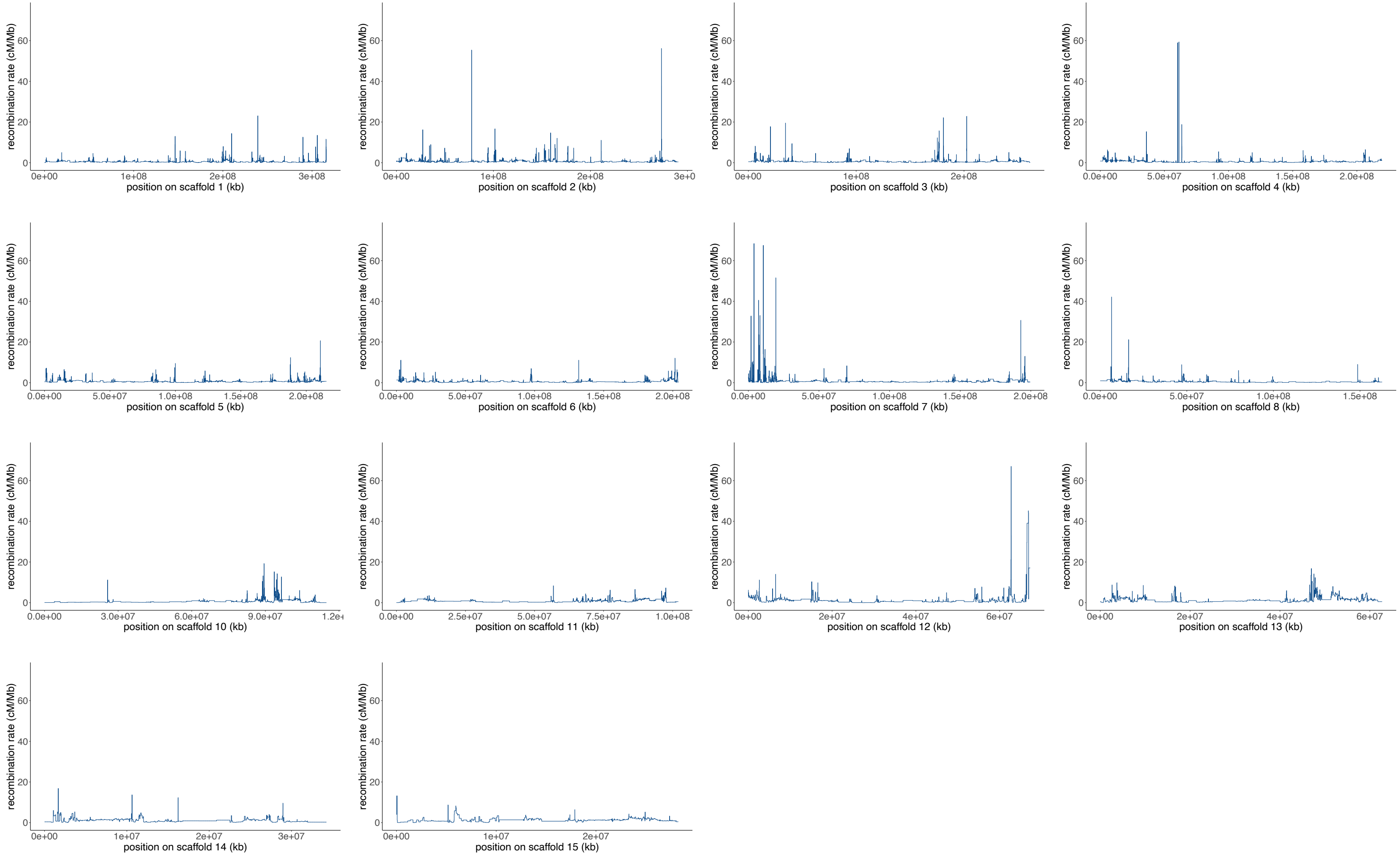

Supplementary Figure S5: Fine-scale recombination rates along each autosomal scaffold for genomic windows of size 1kb.

Supplementary Figure S6

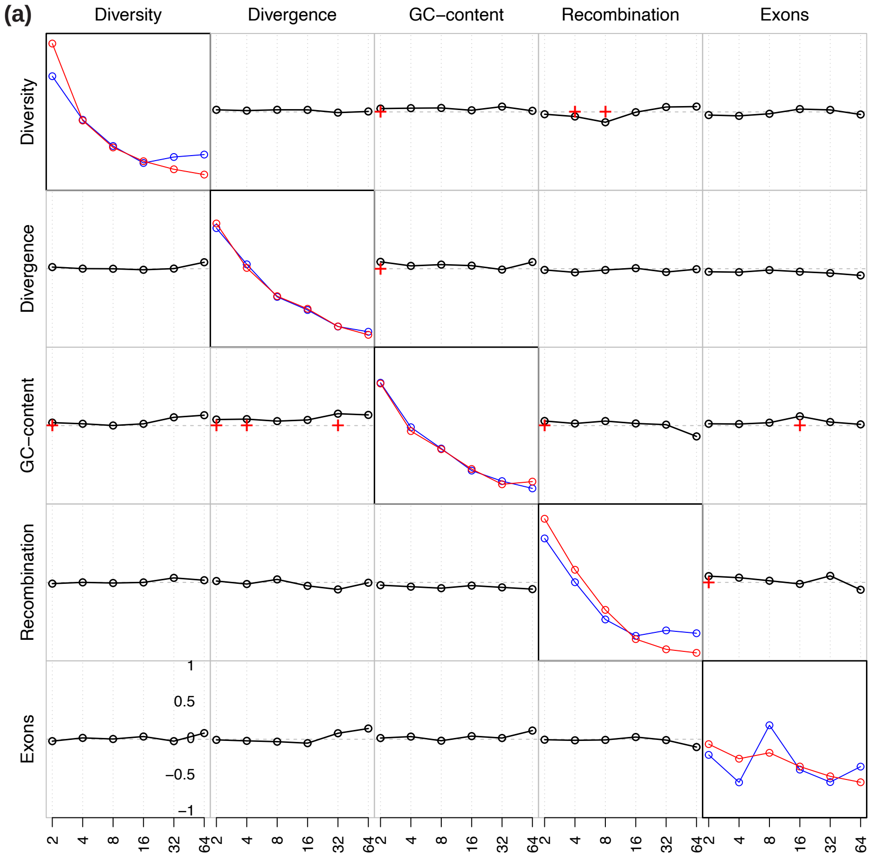

(b)

left-half

	2 kb	4 kb	8 kb	16 kb	32 kb	64 kb
Exons	1.23	2.63	0.55	0.1	0.01	0.16
GC−content	15.26	7.41	3.29	1.32	0.45	0.46
Divergence	0.81	0.35	0.81	0.45	0.15	0.23
Diversity	1.29	0.37	0.18	0.19	0.94	0.62
Adj r^2	0.03	0.04	0.02	0.01	0.01	0.03

right-half

	2 kb	4 kb	8 kb	16 kb	32 kb	64 kb
Exons	0.45	0.83	0.74	0.29	0.29	0.61
GC−content	1.44	0.17	0.25	0.3	0.07	0.65
Divergence	0.51	0.53	0.11	0.05	0.04	0.03
Diversity	0.08	0.7	4.6	0.22	0.86	0.39
Adj r^2	0	0	0.03	0.01	0.01	0.01

Supplementary Figure S6: (a) The detail coefficients of each genomic feature (diagonal plots) on the left and right halves of scaffold 1 (shown in blue and red, respectively) as well as their pairwise correlations based on Kendall's rank correlation (off-diagonal plots with the bottom left showing the left-half and the top right showing the right-half of the scaffold) at a range of (2^n) scales. Correlations significant at the 1%-level under a two-tailed test are highlighted by crosses. (b) Linear model analysis of the detail coefficients. Red and blue coloring indicate significant positive and negative relationships under a two-sided *t*-test, with the color intensity being proportional to the significance level. Adjusted r^2 (Adj r^2) specifies the proportion of heterogeneity that can be explained by the linear model.

Supplementary Figure S7

(a)

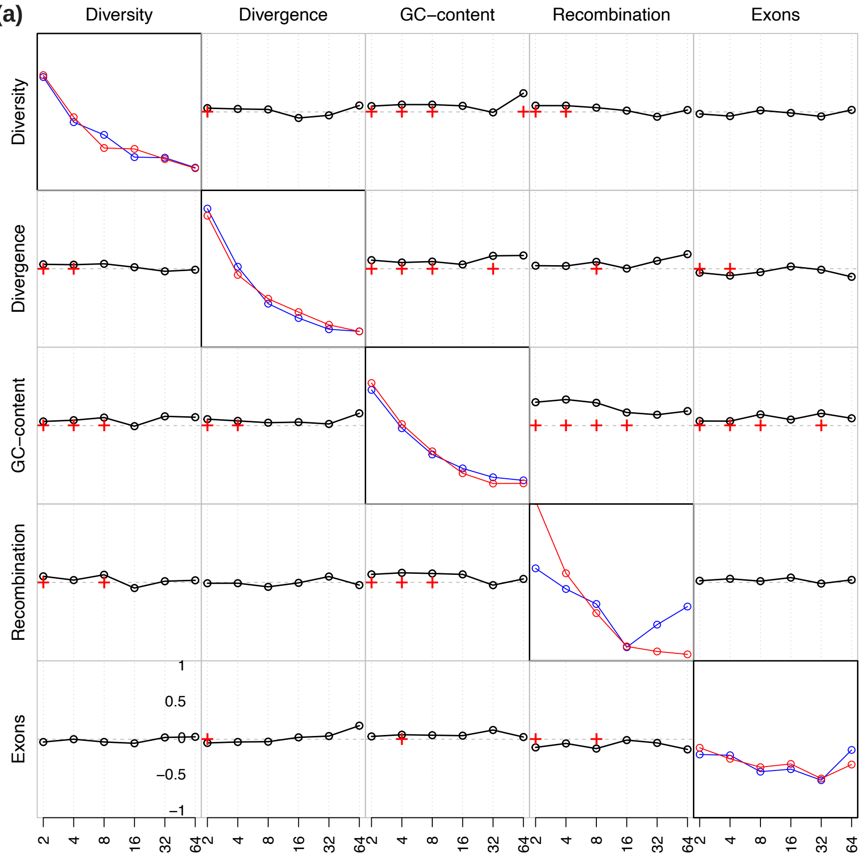

(b)

left-half

	2 kb	4 kb	8 kb	16 kb	32 kb	64 kb
Exons	7.34	0.62	2.55	0.51	0.32	1.24
GC−content	19.9	8.93	4.42	2.23	0.06	0.07
Divergence	2.27	0.12	2.84	0.24	1.2	0.29
Diversity	8.04	1.23	1.47	0.96	0.13	0.02
Adj r^2	0.07	0.04	0.07	0.03	0	0

right-half

	2 kb	4 kb	8 kb	16 kb	32 kb	64 kb
Exons	3.61	1.09	1.49	0.9	0.41	0.09
GC−content	97.45	52.26	21.78	4	1.46	0.29
Divergence	0.11	0.06	1.49	0.3	0.37	0.08
Diversity	7.43	1.95	0.25	0.01	0.54	0.26
Adj r^2	0.22	0.22	0.19	0.05	0.03	0.04

Supplementary Figure S7: (a) The detail coefficients of each genomic feature (diagonal plots) on the left and right halves of scaffold 2 (shown in blue and red, respectively) as well as their pairwise correlations based on Kendall's rank correlation (off-diagonal plots with the bottom left showing the left-half and the top right showing the right-half of the scaffold) at a range of (2^n) scales. Correlations significant at the 1%-level under a two-tailed test are highlighted by crosses. (b) Linear model analysis of the detail coefficients. Red and blue coloring indicate significant positive and negative relationships under a two-sided *t*-test, with the color intensity being proportional to the significance level. Adjusted r^2 (Adj r^2) specifies the proportion of heterogeneity that can be explained by the linear model.

Supplementary Figure S8

(a)

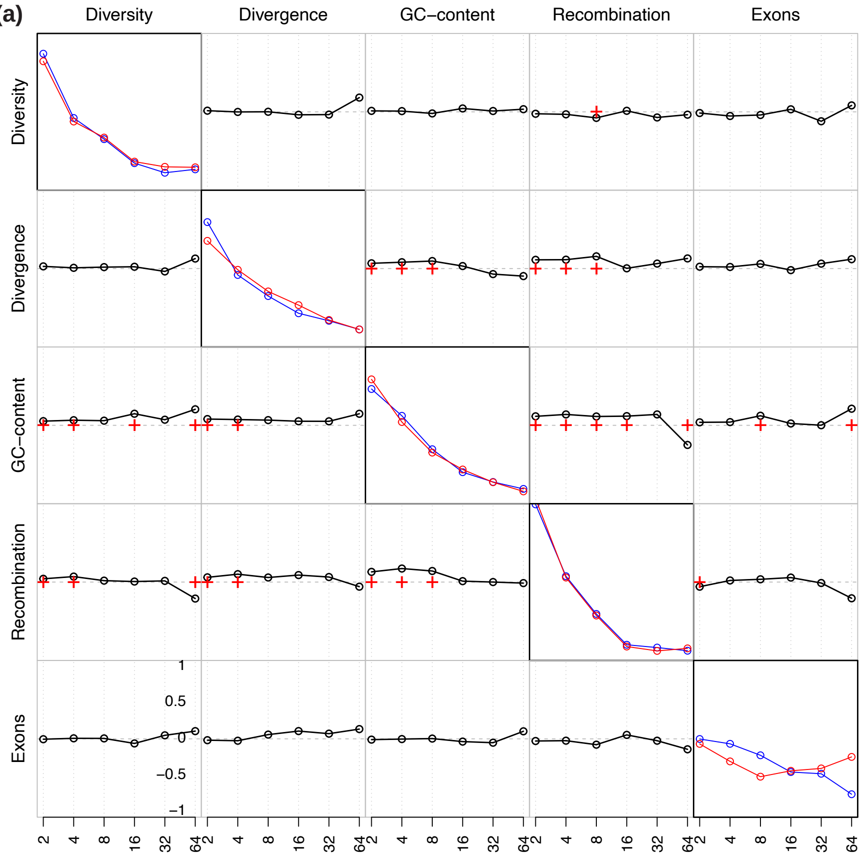

(b)

left-half

	2 kb	4 kb	8 kb	16 kb	32 kb	64 kb
Exons	0.4	1.26	0.96	0.35	0.04	0.03
GC–content	18.11	8.85	5.87	0.49	0.46	1.07
Divergence	1.28	2.42	0.27	1.76	0.75	0.21
Diversity	0.14	0.71	0.01	0.31	0.24	4.97
Adj r^2	0.04	0.05	0.04	0.01	0.01	0.25

right-half

	2 kb	4 kb	8 kb	16 kb	32 kb	64 kb
Exons	1.19	0.7	0.05	0.35	0.13	0.26
GC–content	7.52	8.51	2.32	3.23	1.95	0.19
Divergence	8.39	3.01	2.86	0.29	0.87	0.74
Diversity	2.2	1.48	2.44	0.57	0.46	0.3
Adj r^2	0.04	0.05	0.05	0.03	0.04	0.02

Supplementary Figure S8: (a) The detail coefficients of each genomic feature (diagonal plots) on the left and right halves of scaffold 3 (shown in blue and red, respectively) as well as their pairwise correlations based on Kendall's rank correlation (off-diagonal plots with the bottom left showing the left-half and the top right showing the right-half of the scaffold) at a range of (2^n) scales. Correlations significant at the 1%-level under a two-tailed test are highlighted by crosses. (b) Linear model analysis of the detail coefficients. Red and blue coloring indicate significant positive and negative relationships under a two-sided *t*-test, with the color intensity being proportional to the significance level. Adjusted r^2 (Adj r^2) specifies the proportion of heterogeneity that can be explained by the linear model.

Supplementary Figure S9

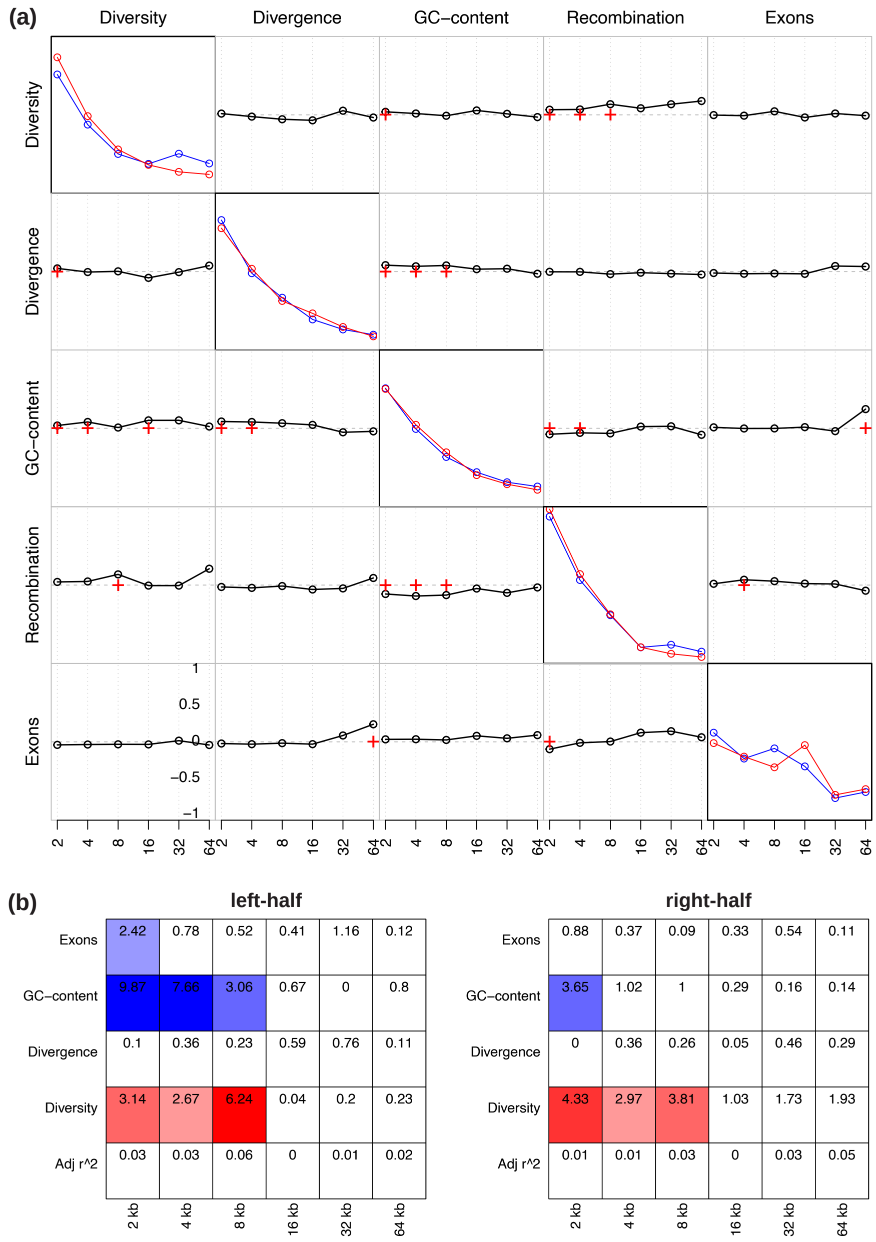

Supplementary Figure S9: (a) The detail coefficients of each genomic feature (diagonal plots) on the left and right halves of scaffold 4 (shown in blue and red, respectively) as well as their pairwise correlations based on Kendall's rank correlation (off-diagonal plots with the bottom left showing the left-half and the top right showing the right-half of the scaffold) at a range of (2^n) scales. Correlations significant at the 1%-level under a two-tailed test are highlighted by crosses. (b) Linear model analysis of the detail coefficients. Red and blue coloring indicate significant positive and negative relationships under a two-sided *t*-test, with the color intensity being proportional to the significance level. Adjusted r^2 (Adj r^2) specifies the proportion of heterogeneity that can be explained by the linear model.

Supplementary Figure S10

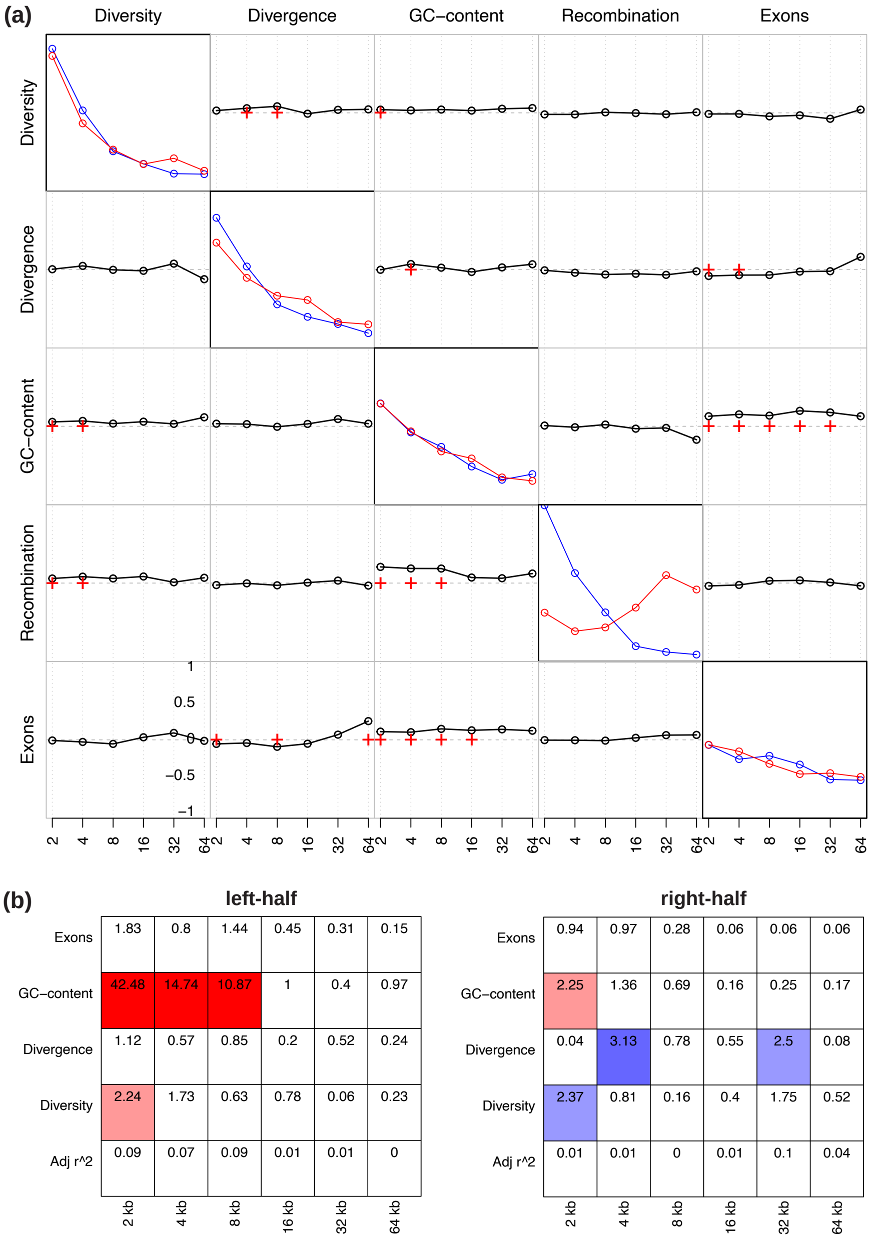

Supplementary Figure S10: (a) The detail coefficients of each genomic feature (diagonal plots) on the left and right halves of scaffold 5 (shown in blue and red, respectively) as well as their pairwise correlations based on Kendall's rank correlation (off-diagonal plots with the bottom left showing the left-half and the top right showing the right-half of the scaffold) at a range of (2^n) scales. Correlations significant at the 1%-level under a two-tailed test are highlighted by crosses. (b) Linear model analysis of the detail coefficients. Red and blue coloring indicate significant positive and negative relationships under a two-sided *t*-test, with the color intensity being proportional to the significance level. Adjusted r^2 (Adj r^2) specifies the proportion of heterogeneity that can be explained by the linear model.

Supplementary Figure S11

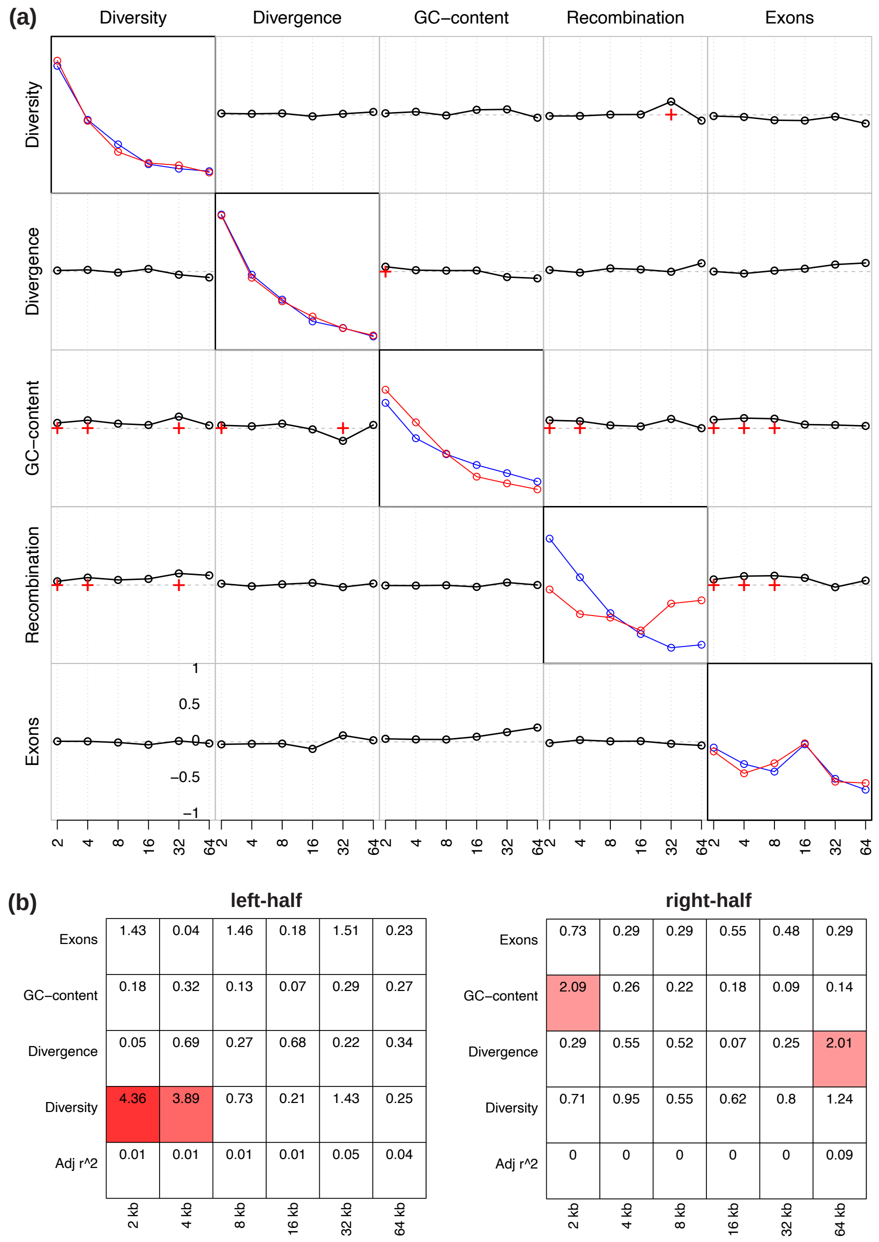

Supplementary Figure S11: (a) The detail coefficients of each genomic feature (diagonal plots) on the left and right halves of scaffold 6 (shown in blue and red, respectively) as well as their pairwise correlations based on Kendall's rank correlation (off-diagonal plots with the bottom left showing the left-half and the top right showing the right-half of the scaffold) at a range of (2^n) scales. Correlations significant at the 1%-level under a two-tailed test are highlighted by crosses. (b) Linear model analysis of the detail coefficients. Red and blue coloring indicate significant positive and negative relationships under a two-sided *t*-test, with the color intensity being proportional to the significance level. Adjusted r^2 (Adj r^2) specifies the proportion of heterogeneity that can be explained by the linear model.

Supplementary Figure S12

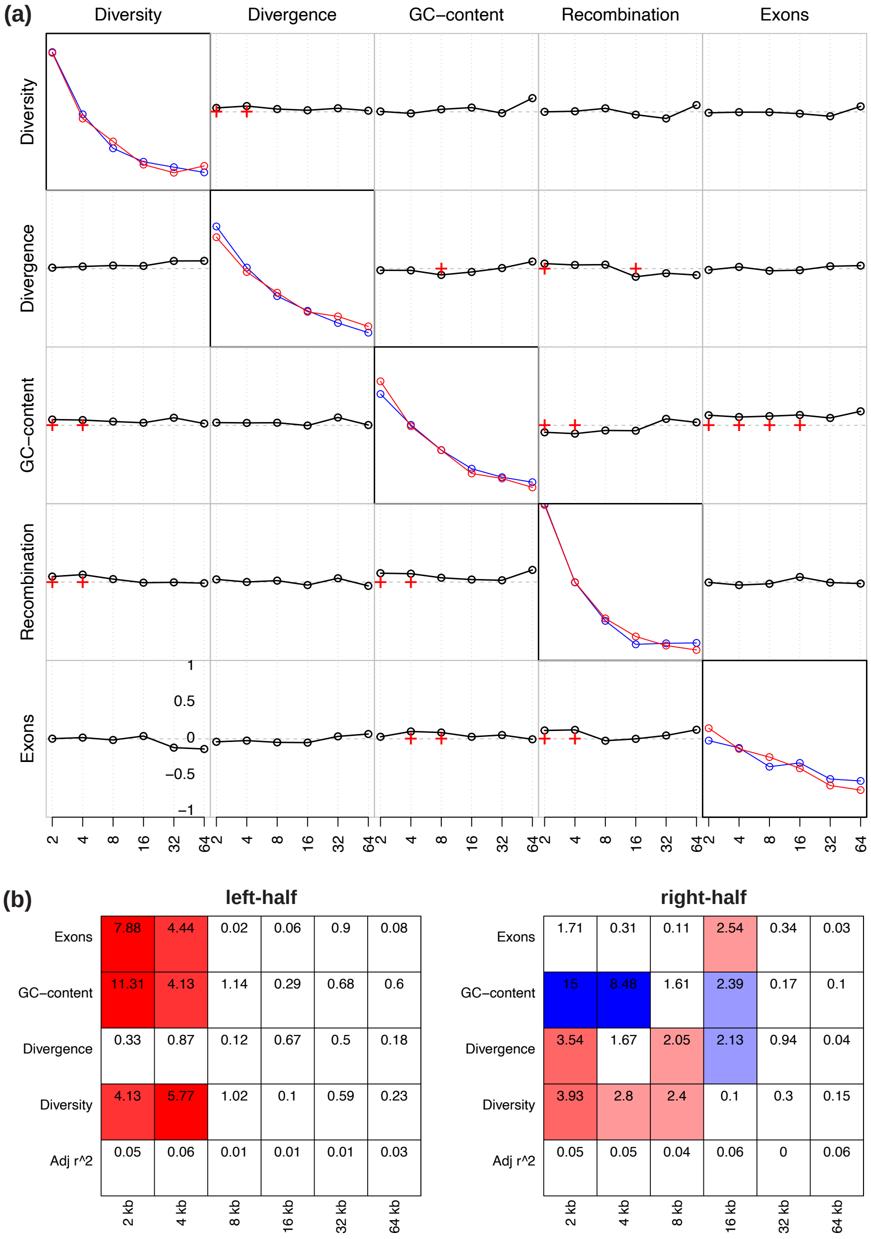

Supplementary Figure S12: (a) The detail coefficients of each genomic feature (diagonal plots) on the left and right halves of scaffold 7 (shown in blue and red, respectively) as well as their pairwise correlations based on Kendall's rank correlation (off-diagonal plots with the bottom left showing the left-half and the top right showing the right-half of the scaffold) at a range of (2^n) scales. Correlations significant at the 1%-level under a two-tailed test are highlighted by crosses. (b) Linear model analysis of the detail coefficients. Red and blue coloring indicate significant positive and negative relationships under a two-sided *t*-test, with the color intensity being proportional to the significance level. Adjusted r^2 (Adj r^2) specifies the proportion of heterogeneity that can be explained by the linear model.

Supplementary Figure S13

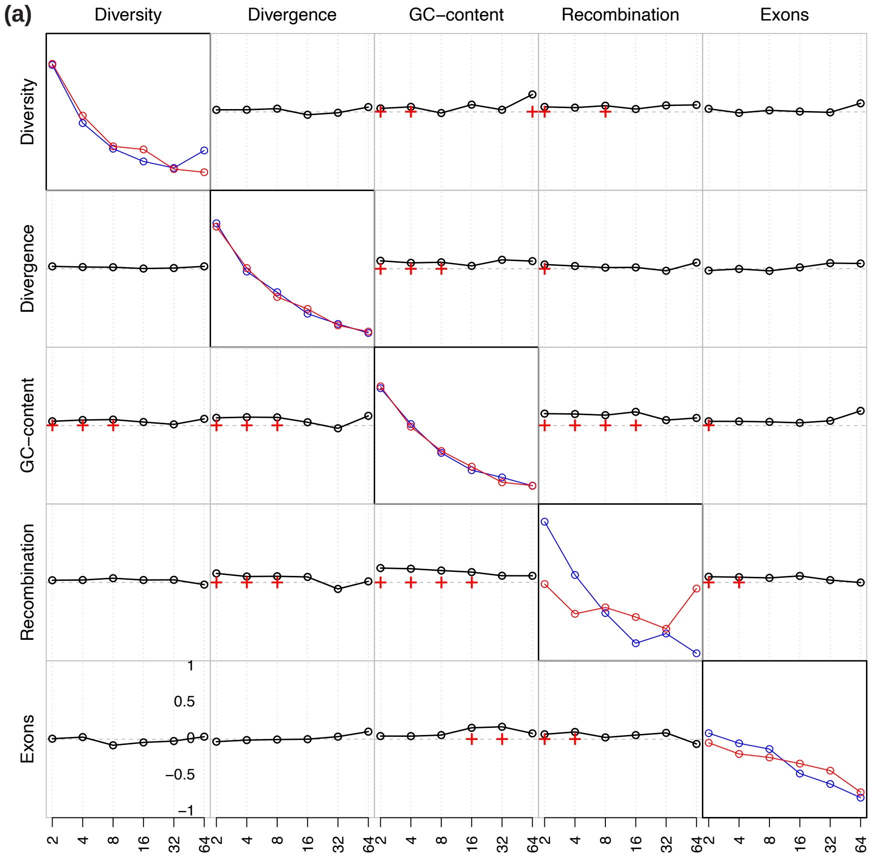

(b)

left-half

	2 kb	4 kb	8 kb	16 kb	32 kb	64 kb
Exons	0.06	0.04	0.02	0.12	0.02	0.36
GC−content	41.1	22.91	6	1.7	0.63	0.42
Divergence	3.05	0.66	0.79	0.59	1.56	0.06
Diversity	0.35	0.05	0.5	0.03	0.42	0.23
Adj r^2	0.1	0.1	0.05	0.01	0.02	0.03

right-half

	2 kb	4 kb	8 kb	16 kb	32 kb	64 kb
Exons	1.24	0.05	0.2	0.29	1.28	0.12
GC−content	24.86	16.1	7.7	3.14	0.66	0.11
Divergence	2.64	0.39	0.05	0.16	1.35	1.89
Diversity	3.82	0.51	1.83	0.12	0.73	0.34
Adj r^2	0.07	0.07	0.06	0.03	0.06	0.04

Supplementary Figure S13: (a) The detail coefficients of each genomic feature (diagonal plots) on the left and right halves of scaffold 8 (shown in blue and red, respectively) as well as their pairwise correlations based on Kendall's rank correlation (off-diagonal plots with the bottom left showing the left-half and the top right showing the right-half of the scaffold) at a range of (2^n) scales. Correlations significant at the 1%-level under a two-tailed test are highlighted by crosses. (b) Linear model analysis of the detail coefficients. Red and blue coloring indicate significant positive and negative relationships under a two-sided *t*-test, with the color intensity being proportional to the significance level. Adjusted r^2 (Adj r^2) specifies the proportion of heterogeneity that can be explained by the linear model.

Supplementary Figure S14

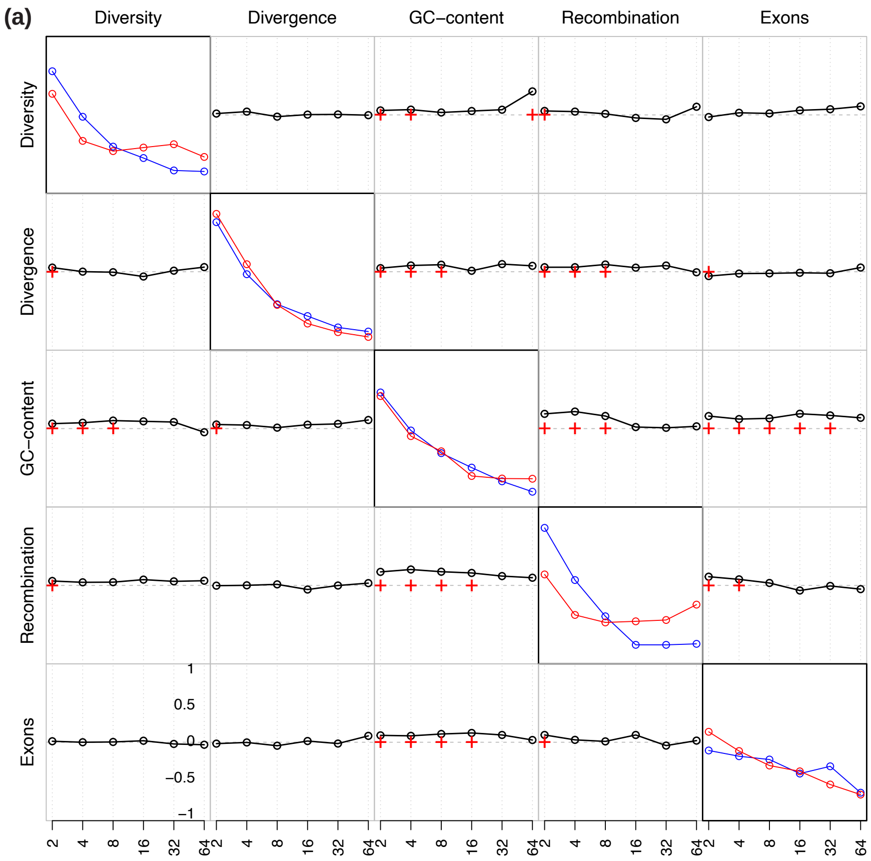

(b)

left-half

	2 kb	4 kb	8 kb	16 kb	32 kb	64 kb
Exons	5.47	0.67	0.16	0.58	0.21	0.02
GC−content	32.59	19.27	9.67	5.16	1.33	0.57
Divergence	0.84	0.25	0.25	0.61	0.05	1.07
Diversity	1.41	0.79	0.93	0.32	0.54	0.91
Adj r^2	0.09	0.08	0.08	0.08	0.02	0.02

right-half

	2 kb	4 kb	8 kb	16 kb	32 kb	64 kb
Exons	0.97	0.07	0.13	1.44	0.33	0.54
GC−content	35.55	23.13	5.8	0.68	0.45	0.31
Divergence	1.09	0.53	1.15	0.07	0.45	0.08
Diversity	2.9	0.86	1.01	0.27	3.92	1.38
Adj r^2	0.09	0.1	0.05	0.01	0.1	0.04

Supplementary Figure S14: (a) The detail coefficients of each genomic feature (diagonal plots) on the left and right halves of scaffold 10 (shown in blue and red, respectively) as well as their pairwise correlations based on Kendall's rank correlation (off-diagonal plots with the bottom left showing the left-half and the top right showing the right-half of the scaffold) at a range of (2^n) scales. Correlations significant at the 1%-level under a two-tailed test are highlighted by crosses. (b) Linear model analysis of the detail coefficients. Red and blue coloring indicate significant positive and negative relationships under a two-sided *t*-test, with the color intensity being proportional to the significance level. Adjusted r^2 (Adj r^2) specifies the proportion of heterogeneity that can be explained by the linear model.

Supplementary Figure S15

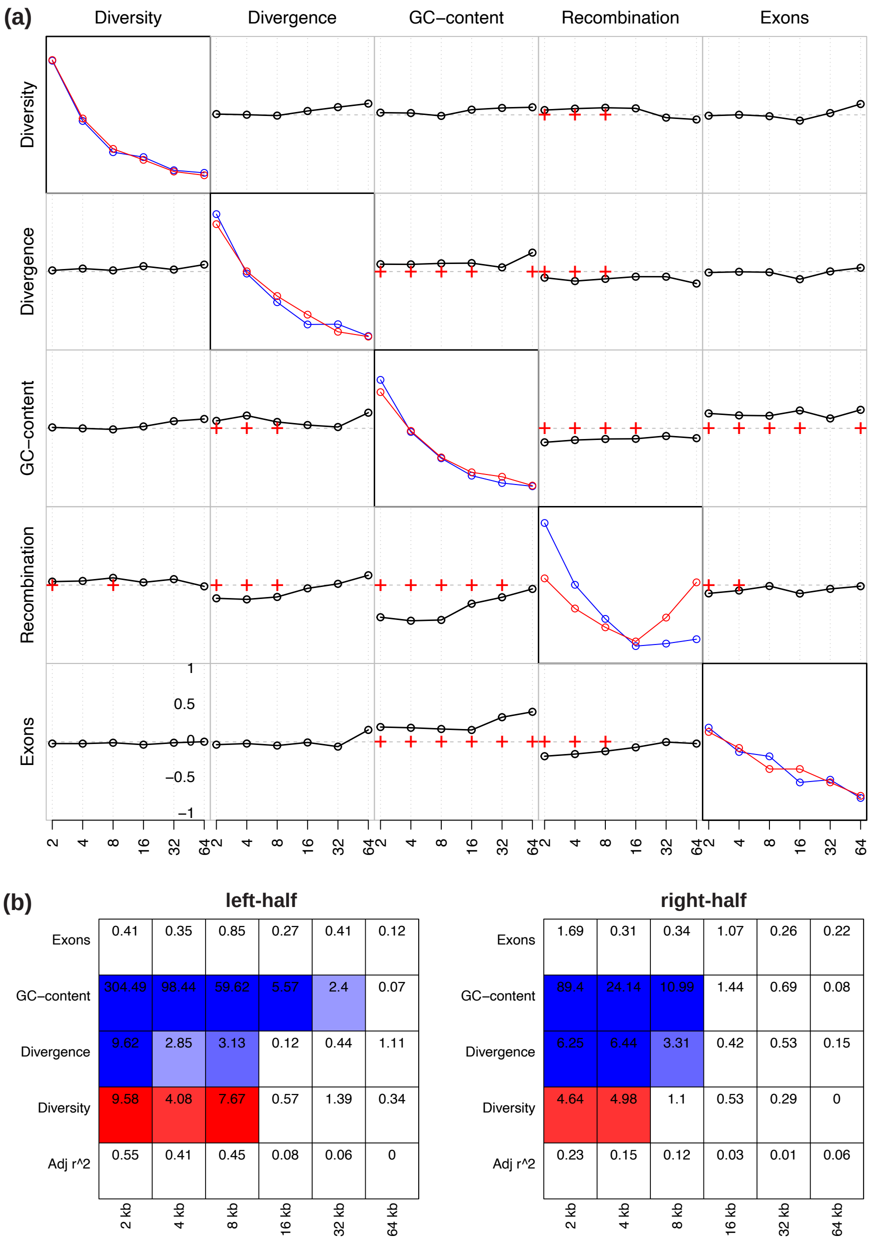

Supplementary Figure S15: (a) The detail coefficients of each genomic feature (diagonal plots) on the left and right halves of scaffold 11 (shown in blue and red, respectively) as well as their pairwise correlations based on Kendall's rank correlation (off-diagonal plots with the bottom left showing the left-half and the top right showing the right-half of the scaffold) at a range of (2^n) scales. Correlations significant at the 1%-level under a two-tailed test are highlighted by crosses. (b) Linear model analysis of the detail coefficients. Red and blue coloring indicate significant positive and negative relationships under a two-sided *t*-test, with the color intensity being proportional to the significance level. Adjusted r^2 (Adj r^2) specifies the proportion of heterogeneity that can be explained by the linear model.

Supplementary Figure S16

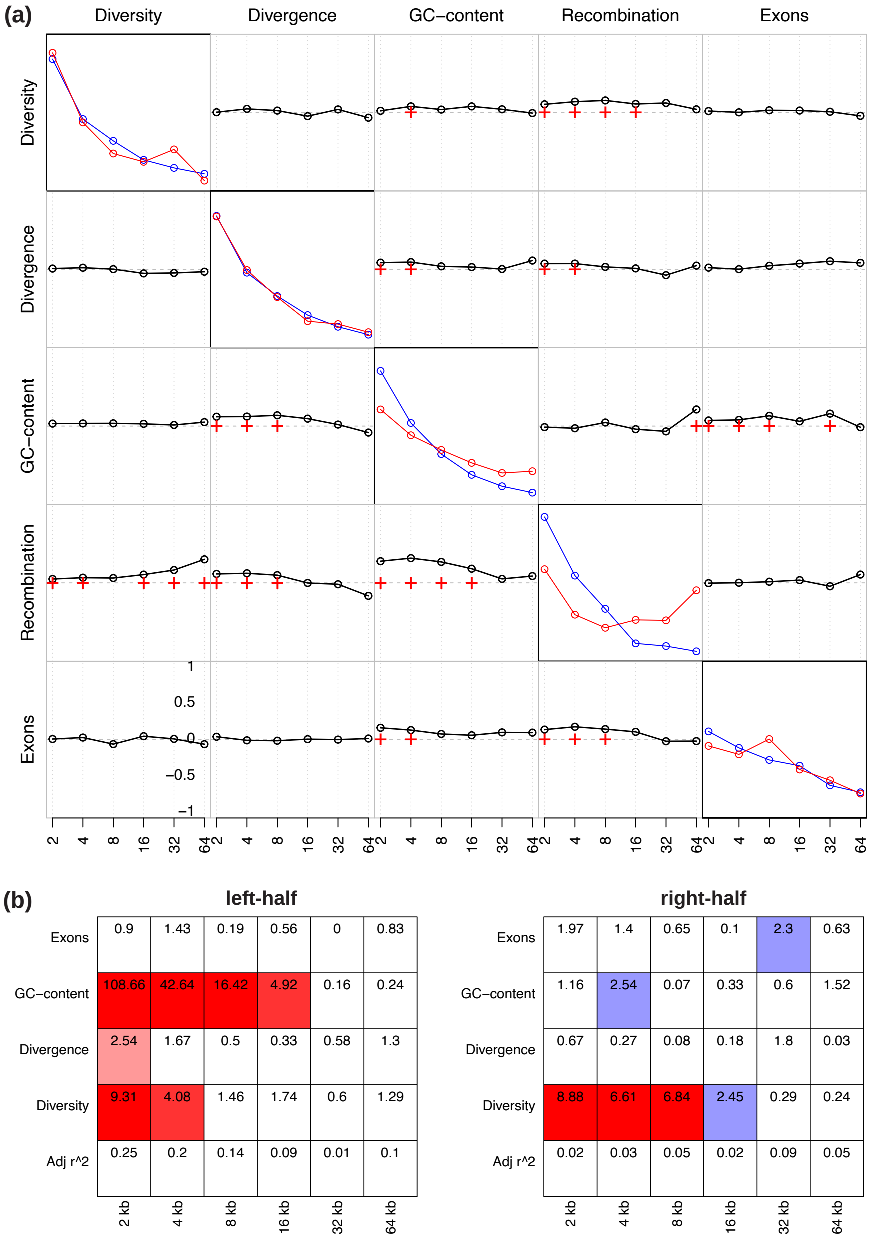

Supplementary Figure S16: (a) The detail coefficients of each genomic feature (diagonal plots) on the left and right halves of scaffold 12 (shown in blue and red, respectively) as well as their pairwise correlations based on Kendall's rank correlation (off-diagonal plots with the bottom left showing the left-half and the top right showing the right-half of the scaffold) at a range of (2^n) scales. Correlations significant at the 1%-level under a two-tailed test are highlighted by crosses. (b) Linear model analysis of the detail coefficients. Red and blue coloring indicate significant positive and negative relationships under a two-sided *t*-test, with the color intensity being proportional to the significance level. Adjusted r^2 (Adj r^2) specifies the proportion of heterogeneity that can be explained by the linear model.

Supplementary Figure S17

(a)

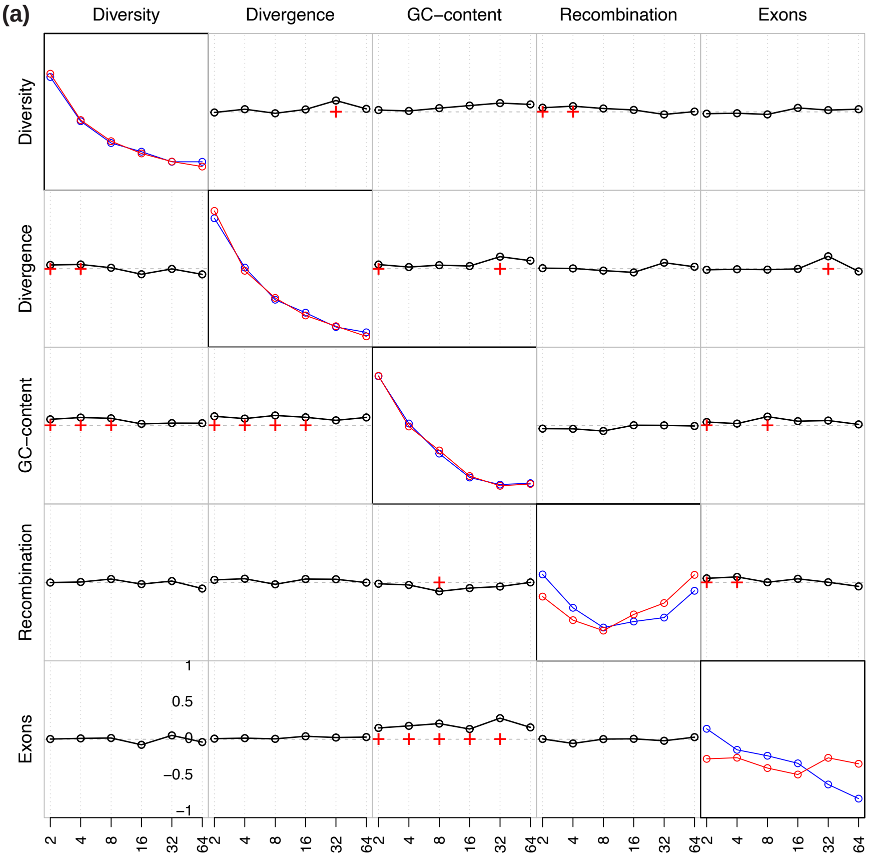

(b)

left-half

	2 kb	4 kb	8 kb	16 kb	32 kb	64 kb
Exons	1.8	0.2	1.06	0.6	1.61	1.35
GC−content	83.03	34.02	19.75	2.72	2.15	0.29
Divergence	0.23	0.61	0.02	0.29	0.03	2.52
Diversity	0.2	0.1	2.68	0	0.03	0.16
Adj r^2	0.18	0.14	0.16	0.03	0.05	0.14

right-half

	2 kb	4 kb	8 kb	16 kb	32 kb	64 kb
Exons	6.51	2.43	0.02	0.92	0.07	0.15
GC−content	4.24	2.36	2.85	0.58	0.54	0.2
Divergence	2.93	1.98	1.14	0.68	0.41	0.25
Diversity	3.59	4.33	1.93	0.16	0.02	0.4
Adj r^2	0.03	0.03	0.03	0.01	0.02	0.04

Supplementary Figure S17: (a) The detail coefficients of each genomic feature (diagonal plots) on the left and right halves of scaffold 13 (shown in blue and red, respectively) as well as their pairwise correlations based on Kendall's rank correlation (off-diagonal plots with the bottom left showing the left-half and the top right showing the right-half of the scaffold) at a range of (2^n) scales. Correlations significant at the 1%-level under a two-tailed test are highlighted by crosses. (b) Linear model analysis of the detail coefficients. Red and blue coloring indicate significant positive and negative relationships under a two-sided *t*-test, with the color intensity being proportional to the significance level. Adjusted r^2 (Adj r^2) specifies the proportion of heterogeneity that can be explained by the linear model.

Supplementary Figure S18

(a)

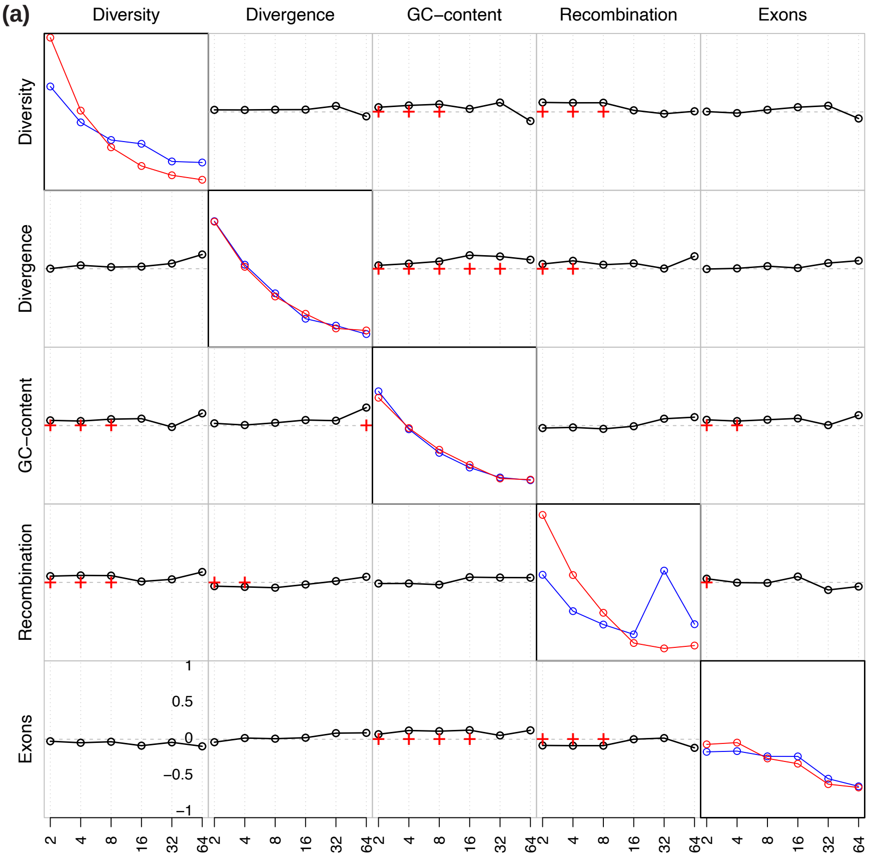

(b)

left-half

	2 kb	4 kb	8 kb	16 kb	32 kb	64 kb
Exons	10.13	2.78	2.17	0.16	0.24	0.27
GC−content	0.38	0.39	0.74	0.68	0.04	0.63
Divergence	2.55	2.81	0.64	0.49	0.7	0.08
Diversity	10.83	6.62	0.89	0.77	0.03	0.08
Adj r^2	0.05	0.04	0.02	0	0.02	0.03

right-half

	2 kb	4 kb	8 kb	16 kb	32 kb	64 kb
Exons	0.08	0.42	0.25	0.78	1.08	0.72
GC−content	5.78	1.54	1.93	0.27	0.63	0.59
Divergence	3.42	3.14	1.51	1.2	0.16	1.03
Diversity	19.56	11.1	7.35	0.07	0.74	0
Adj r^2	0.05	0.05	0.06	0.01	0.02	0.05

Supplementary Figure S18: (a) The detail coefficients of each genomic feature (diagonal plots) on the left and right halves of scaffold 14 (shown in blue and red, respectively) as well as their pairwise correlations based on Kendall's rank correlation (off-diagonal plots with the bottom left showing the left-half and the top right showing the right-half of the scaffold) at a range of (2^n) scales. Correlations significant at the 1%-level under a two-tailed test are highlighted by crosses. (b) Linear model analysis of the detail coefficients. Red and blue coloring indicate significant positive and negative relationships under a two-sided *t*-test, with the color intensity being proportional to the significance level. Adjusted r^2 (Adj r^2) specifies the proportion of heterogeneity that can be explained by the linear model.

Supplementary Figure S19

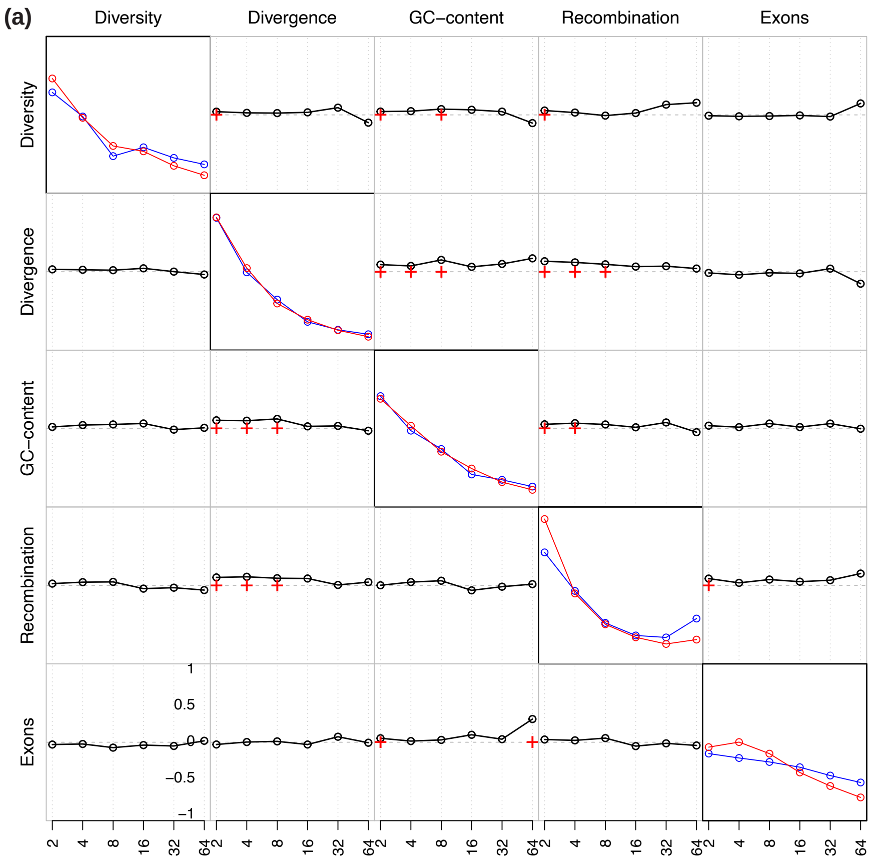

(b)

left-half

	2 kb	4 kb	8 kb	16 kb	32 kb	64 kb
Exons	0.39	0.37	1.89	0.37	0.37	0.01
GC–content	0.84	1.84	0.75	0.76	0.5	0.25
Divergence	11.68	3.36	2.8	2.06	0.9	0.06
Diversity	0.47	1.28	0.73	0.26	0.32	0.21
Adj r^2	0.03	0.02	0.03	0.02	0	0.05

right-half

	2 kb	4 kb	8 kb	16 kb	32 kb	64 kb
Exons	2.88	1.7	0.36	0.28	0.54	0.23
GC–content	4.6	3.18	0.72	0.08	1.25	0.25
Divergence	11.4	3.35	1.01	1.48	0.25	0.44
Diversity	3	2.42	0.16	0.85	0.82	1.35
Adj r^2	0.05	0.04	0	0.01	0.03	0.02

Supplementary Figure S19: (a) The detail coefficients of each genomic feature (diagonal plots) on the left and right halves of scaffold 15 (shown in blue and red, respectively) as well as their pairwise correlations based on Kendall's rank correlation (off-diagonal plots with the bottom left showing the left-half and the top right showing the right-half of the scaffold) at a range of (2^n) scales. Correlations significant at the 1%-level under a two-tailed test are highlighted by crosses. (b) Linear model analysis of the detail coefficients. Red and blue coloring indicate significant positive and negative relationships under a two-sided *t*-test, with the color intensity being proportional to the significance level. Adjusted r^2 (Adj r^2) specifies the proportion of heterogeneity that can be explained by the linear model.

Supplementary Figure S20

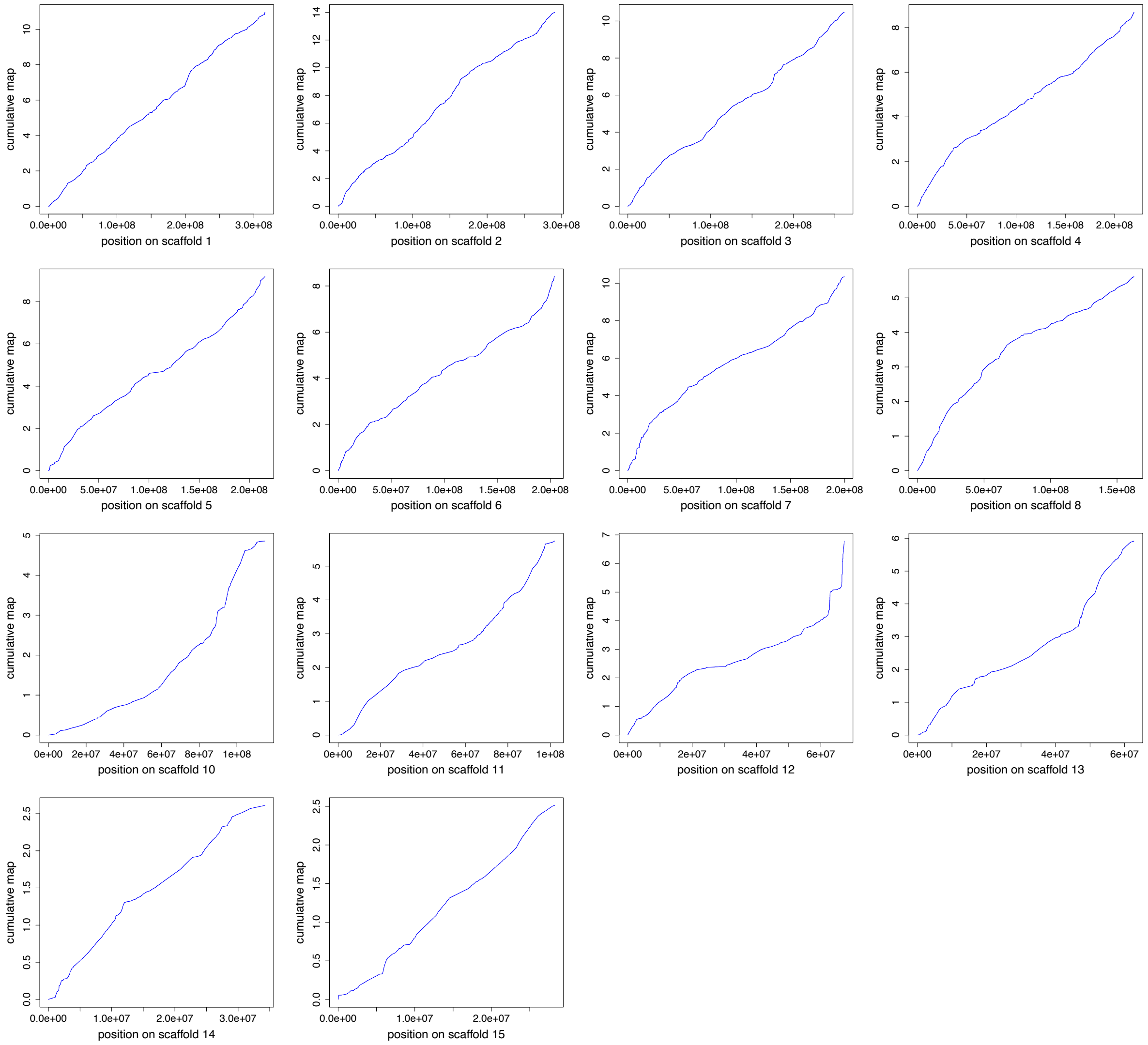

Supplementary Figure S20: Cumulative map of each autosomal scaffold.
